# Quantifying racial disparities in media representations of gun violence at scale

**DOI:** 10.1073/pnas.2505499123

**Published:** 2026-01-16

**Authors:** Ruth Bagley, Susan Burtner, Andrew V. Papachristos, Rob Voigt

**Affiliations:** ^a^Department of Linguistics, Northwestern University, Evanston, IL 60208; ^b^Center for Neighborhood Engaged Research & Science, Northwestern University, Evanston, IL 60208; ^c^Institute for Policy Research, Northwestern University, Evanston, IL 60208; ^d^Center for Firearm Injury Prevention, University of Washington, Seattle, WA 98195; ^e^Department of Pediatrics, University of Washington, Seattle, WA 98195; ^f^Department of Sociology, Northwestern University, Evanston, IL 60208; ^g^Department of Linguistics, University of California, Davis, CA 95616

**Keywords:** gun violence, racial disparities, media representation, natural language processing

## Abstract

Previous research has documented racial disparities in gun violence news coverage in limited and small-scale contexts. This study curates and analyzes a large-scale dataset of news articles linked to specific incidents of gun violence to test for systematic race-related differences in representation across the US news media. Using computational techniques, we quantify how much media attention an incident gets, the topics and linguistic style of articles, and how participants in the incidents are framed. We find significant generalized disparities in media coverage and portrayal of incidents depending on whether they occur in neighborhoods that are majority white or majority people of color (POC), including increased media attention on police shootings if they occur in majority POC neighborhoods, greater focus on the people involved in incidents in majority white neighborhoods, and increased racialization and framing related to crime in majority POC neighborhoods.

## Significance

Media reports on incidents of gun violence differ significantly when the incident occurs in a majority white or majority people of color neighborhood, even after controlling for characteristics of the incident and neighborhood. We use computational linguistic methods to analyze over 35,000 articles, and introduce a methodology for analyzing both high-level and more nuanced trends in media reports. Our findings provide empirical evidence that there are disparities in how incidents are covered and framed in the news that echo certain racial stereotypes around crime and are associated with the racial composition of the neighborhood where they occur.

Gun violence in the US disproportionately affects communities of color. According to data from the Gun Violence Archive (1), 70% of the over 46,000 gun violence incidents in 2022 that resulted in injury or death occurred in neighborhoods where the majority of residents are people of color (POC), despite POC comprising only 38.4% of the US population (2). Media coverage of such incidents can perpetuate harmful biases, extending the impact beyond immediate trauma.

On a personal level, many victims of gun violence state that they have felt dehumanized or retraumatized by the way the news reported on the event (3). For instance, when Tamir Rice was shot and killed by police in 2014, some news outlets referred to 12 y old Rice as a “Black male with gun” rather than a boy playing with a toy gun (4); other coverage mentioned the criminal records of Tamir’s father and mother, information that felt irrelevant and insensitive to many readers (5). On a broader societal level, evidence suggests that media coverage of these incidents can impact public support for policies to address gun violence, as well as perpetuate racial biases (6–9). In this work, we propose a systematic approach to understand disparities in coverage at scale, using a large dataset sourced from news publications throughout the United States to analyze the role of race in both which incidents the media chooses to cover and how those incidents are portrayed.

Existing research shows that the distribution of coverage of incidents of gun violence in America paints a nonrepresentative picture of the issue, which can lead to inaccurate public perceptions. For instance, mass shootings get significantly more media attention than other types of gun violence (10–12), and when surveyed, 25% of American adults believed mass shootings were the biggest cause of gun deaths, even though 60% of gun deaths are suicides and only 3% of gun deaths are from mass shootings (13). In addition, shootings by minors and school shootings are more likely to be covered if the shooter was white (14, 15). Urban gun violence often occurs in small social networks and geographic areas (16), and community-level data have been used to show that incidents in neighborhoods with greater income inequality and racialized economic segregation are underreported (10).

Race-related disparities also extend to the language used to cover incidents. Content analysis of media portrayals of gun violence has used human annotation to target particular linguistic strategies and identify race-related differences in the portrayals of participants—both shooters and victims. In mass shootings, Black and Muslim shooters are often represented using race-related stereotypes such as “thugs” or “terrorists” (17), while white shooters are more often framed as mentally ill and are more likely to be portrayed sympathetically (18). News coverage of homicide is also less likely to describe victims in POC neighborhoods as multifaceted, complex people with relationships or social roles beyond the role of victim (19).

Approaches using natural language processing (NLP) techniques on larger scale datasets have been applied to examine trends in the framing of gun violence in the media, particularly in relation to the partisanship of news sources and audiences (20–22). For instance, media coverage of gun violence becomes more politicized and partisan in the immediate aftermath of a major mass shooting (21). NLP technologies have also been used on news articles about gun violence for the purposes of information extraction (23). Data linkage with linguistic analysis at scale has been applied to coverage of police violence (rather than gun violence) to identify differences in media portrayals of police along partisan lines; liberal sources more frequently frame the violence as a systemic issue, while conservative sources more frequently justify the actions of the police (24).

However, these computational studies did not incorporate detailed incident and participant characteristics, and a key issue in identifying linguistic disparities in media depictions of incidents of gun violence has been the linking of media and incident data on a large enough scale to draw generalized conclusions about how gun violence is represented in the media. We address this issue by using computational methods to curate a dataset of 35,991 articles about gun violence linked to specific incidents across the United States occurring in neighborhoods with a 60% majority of white or POC residents (hereafter “majority POC” and “majority white”).

We test our initial hypothesis that there are widespread, quantifiable differences in media depictions relating to race by training a large language model (LLM) to predict whether an article in our dataset describes an incident in a majority POC or majority white neighborhood. We use coarsened exact matching on incident covariates to select a balanced 50-50 split of articles from each neighborhood type, and remove explicit race-related information and person names. In this setting, we find the LLM can predict neighborhood racial composition with 75.9% accuracy, compared to a random guessing baseline of 50%, suggesting there are substantial differences in the content of articles that cover incidents of gun violence (more details in *SI Appendix*, section 6).

In order to understand the precise nature of those differences, we develop computational tools to quantify disparities in coverage, content, and framing related to neighborhood racial composition. We use four metrics of coverage to identify the relationship between incident characteristics and media attention. We also analyze the text and propose an ontology of content and framing features that capture differences in representation in portrayals of gun violence. Content is analyzed through mentions of specific topics and quoted speakers, and framing is explored from two perspectives: explicitly in the linguistic characteristics of the articles and implicitly in words and descriptors closely associated with participants as they are portrayed in articles.

Our analysis provides a comprehensive and contextualized view of the role of race in media portrayals of gun violence, and finds robust associations between media representations and neighborhood racial composition even when controlling for incident- and neighborhood-level covariates. While we are not able to make causal claims, the descriptive analysis presented here both establishes evidence for disparate impact by race in media coverage and representations of gun violence (in the sense of ref. 25) and enumerates a set of specific linguistic behaviors associated with these disparities.

## Results

### Coverage.

Our first step in identifying disparities in media treatment of gun violence is examining incident coverage and media attention. Prior work has often used police data from specific cities for their analyses; while some police departments can provide more comprehensive incident data (12), this is not consistent across departments, and therefore using data from GVA can facilitate a broader analysis of news coverage across the United States. The structure of our dataset allows us to examine four metrics of coverage: the number of articles written about an incident, the average length of those articles, and whether an incident gets national coverage, and whether the incident was covered by more than 1 article.

We fit linear (for article count and length) and logistic (for national coverage and multiple articles) regression models predicting these coverage outcomes from incident characteristics, including whether it was a school shooting, involved gangs or drugs, or resulted in no or many deaths (See *Statistical Analysis* for the full list of features). The predictors also included neighborhood racial composition and interaction effects between neighborhood composition and all incident characteristics. All coverage metrics are predominantly aligned in terms of both coefficient significance and directionality; we therefore use article count as our primary metric to more easily compare our findings to prior work. We find that regardless of neighborhood composition, incidents receive more coverage when they are school shootings (b=6.603, P<0.001), result in at least four deaths (b=12.918, P<0.001), or involve police officers (b=4.601, P<0.001) or assault weapons (b=5.264, P<0.001). Full regression outputs are in *SI Appendix*, Table S4.

**Fig. 1.**
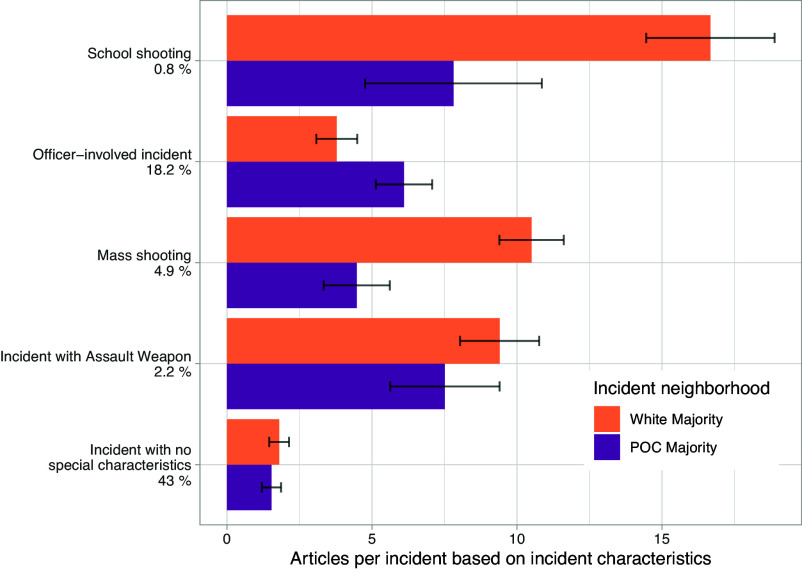
Marginal estimates of the number of articles an incident is likely to receive based on neighborhood demographics and incident characteristics. Error bars represent 95% CIs. Percentages represent the proportion of matched incidents with each characteristic.

We further observe stark disparities in the amount of news coverage incidents receive relative to neighborhood demographics, quantified by interaction effects between incident characteristics and neighborhood type. Fig. 1 shows the number of articles that incidents are likely to receive relative to incident characteristics; mass shootings (b=2.672, P<0.001) and school shootings (b=7.890, P<0.001) are significantly more likely to get more coverage if they occur in majority white neighborhoods. For example, a mass shooting in a majority POC neighborhood will likely have approximately four articles about the incident; an incident with the same characteristics that occurs in a majority white neighborhood is likely to be covered by approximately ten articles. Coverage of officer-involved incidents show the opposite pattern: An incident involving police will receive more coverage if it occurs in a majority POC neighborhood than if it occurs in a majority white neighborhood (b=−2.784, P<0.001).

## Dataset

Using incident data from the Gun Violence Archive (GVA),^*^ news articles from the News on the Web (NOW) corpus (26), and census data (27), we curate a set of 35,991 articles from 2014 to 2023 with extensive metadata, each covering exactly one incident of gun violence in the United States occurring within one week of the article’s publication. Each article is also classified as covering an incident in a census tract (“neighborhood”) that is majority white (≥60% non-Hispanic white population) or majority POC (≤40% non-Hispanic white population). 7,873 additional articles were matched to incidents that occurred in mixed neighborhoods (between 40 and 60% non-Hispanic white), but not used in the main analyses (see *SI Appendix*, section 6 for analyses including the full set of articles).

### Text Analysis.

To get a more complete picture of the disparities in media portrayals of incidents of gun violence, we analyze the language in the articles, both what the articles say (“content”) and also how they say it (“framing”).

To develop a methodology for interpretable linguistic analysis in this domain, we first develop algorithms for extracting a set of quantifiable linguistic features that appear in articles, and examine associations with the individual features in an exploratory manner. We then curate and propose an ontology of larger conceptual categories into which these features can be grouped. Using regressions predicting racial composition based on each feature group in the ontology as well as incident and neighborhood characteristics, we directly test whether the broader conceptual differences hold at scale and across neighborhoods of varying population density, accounting for confounds and multiple comparisons.

Drawing from topics of focus in prior related work (3, 8, 18, 19), our ontology includes nine key content areas curated to address relevant features in gun violence news. The specific constituent features included in each category are depicted in Table 1. Content features are operationalized as keyword lists (*SI Appendix*, Table S2) which can be counted in news article text, while framing features are measured using preexisting lexicons or models.

**Table 1.** Content and linguistic feature categories used in the analyses

Name	Association	Description	Constituent features
Agency	−0.050**	Measures of how actively vs. passively victims, shooters, and police are framed	Agency of civilian victims, shooters, and the police
Broader narrative	−0.124***	Content connected to the broader issue of gun violence beyond a single incident	Mentions of notable incidents, policy, and the issue of gun violence in general
Complex personhood	0.151***	Extent to which persons are presented as multidimensional and/or part of a community	Mentions of family relationships, mental health, or social roles like student, employee, or friend (19), quotes by participants or their close relations, and framing of victims and shooters as complex people
Criminality	−0.018	Focus on crime, criminals, and law enforcement	Mentions of legal terms, words relating to crime (like “arrest”), frequency of mentioning police, and framing of victims and shooters as criminals
Incident report style	−0.044*	Measures of linguistic and stylistic features that might be associated with a formal incident report	Formality, concreteness, frequency of numbers and statistics, complexity of language, and frequency of nonsubjective language
Mortality	−0.055**	Descriptions of physical harm	Mentions of death and medical language
Participant focus	0.043*	The frequency with which participants are mentioned by name	Mentions of shooters, victims, and police participants involved in the incident
People of authority	0.141***	Mentions or quotes from political figures or members of law enforcement	Quoted police, local politicians, and national politicians, or mentions of police officers or law enforcement officials
Racialization	−0.090***	Use of words relating implicitly or explicitly to race	Mentions of explicit race, implicitly racialized language (like gang), and explicit mentions of bias relating to race, such as “racism” or “supremacist”

Associations represent the strength of association with white neighborhoods (positive) and POC neighborhoods (negative), quantified by the regression coefficients for the grouped features predicting the racial composition group including controls. * is *P* < 0.05.

We use a logistic regression model predicting neighborhood racial composition from article-level features, including controls for neighborhood composition and incident characteristics, and interpret coefficients for each feature as the strength of association with neighborhood racial composition. To provide greater context for other axes of variation, for content features we also use difference of means to quantify differences in how often each feature appears if it does occur in articles about majority POC versus majority white neighborhoods.

#### Content.

**Fig. 2.**
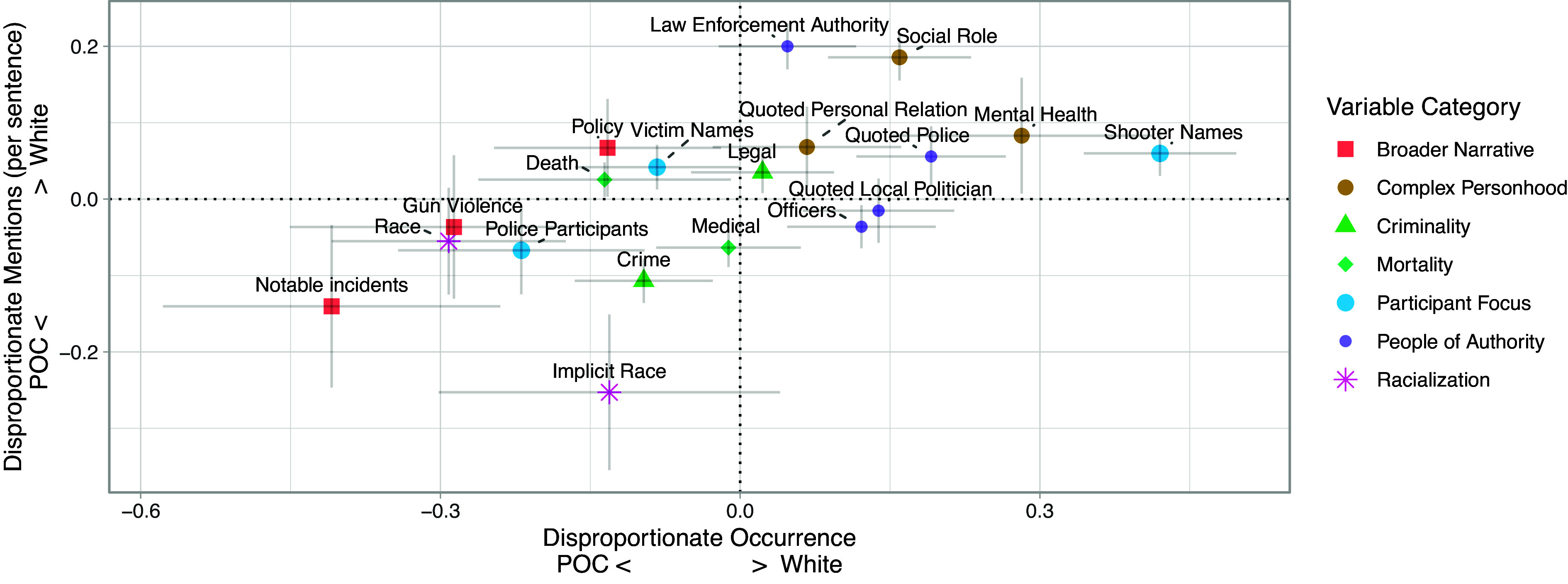
Disproportionate occurrence and mentions per sentence for content features by neighborhood racial composition. Values for disproportionate occurrence (whether a features appears at all) are logistic regression coefficients for each binarized content feature in regressions predicting race binary including controls, and disproportionate mentions per sentence (how often a feature occurs in an article when it appears) is quantified by differences in means between the two groups. Color and shape are used to represent the variable’s category in the ontology. Error bars represent 95% CIs. For readability, the features with no significance on either metric (Family, Bias, and Quoted National Politician) were removed from the graph.

Our findings for content features are shown in Fig. 2. Echoing prior work, we find that language associated with complex personhood (social roles, mental health) is more prevalent for incidents in majority white neighborhoods (18, 19). We also find that features associated with people of authority (mentions of officers or quotes from local politicians or police) are more associated with majority white neighborhoods.

Majority POC neighborhoods on the other hand are more associated with the broader narrative of gun violence (mentions of gun violence itself, policy, and other notable incidents). Explicit mentions of race, such as “white” or “black,” are also strongly associated with majority POC neighborhoods. In addition, we find that descriptions of someone as “white” rarely occur without a description of someone as a race other than white, while the reverse is not true (*SI Appendix*, section 6.D). These findings together suggest that media representations of gun violence tend to reflect a “white as default” perspective, where white is the normative race and therefore race is mainly mentioned when someone is not white (28, 29).

The emphasis on those involved in incidents of gun violence is also a source of variation. The participants in the incidents are divided into three distinct role categories: civilian shooters, civilian victims, and police participants. We find that articles about incidents in majority white neighborhoods are much more likely to mention shooter names. Articles about majority POC neighborhoods are more likely to mention victim names but that does not necessarily mean that those articles focus more on the victims, as we can see that if a victim name appears in an article about a white neighborhood, they are usually mentioned more frequently; this suggests that articles about people of color might more reliably mention a victim’s name at some point but not talk about them in as much depth. We also find that although articles about majority white neighborhoods are more likely to mention police in general (officers), when police are actually involved in incidents, those police participants are mentioned more if that incident occurred in a majority POC neighborhood.

#### Framing.

We measure three categories of framing features: stylistic features, participant agency, and implicit framing.

The stylistic features we implemented are Subjective Language, Readability, Formality, Concreteness, and frequency of Numbers and Statistics, which were measured using preexisting lexicons or off-the-shelf models. Agency is evaluated using syntactic parsing and lists of verbs associated with high and low agency (30) to determine whether participants are portrayed more as agents (with an active role) or patients (with a passive role).

Articles can also vary in terms of implicit framing of participants involved in the incident; for example, if a victim is framed as part of a family, they might be viewed more sympathetically by the public than if they are framed as a criminal. However, framing can often be more subtle, or implicit, than using the words “criminal” or “parent.” Based on previous work, we selected two frames to analyze: if the participant is portrayed as a complex person (19) or a criminal (18). Drawing from prior work on implicit dehumanization (31), we use a large language model to estimate how civilian victims and shooters are implicitly described, or framed, by the article.

**Fig. 3.**
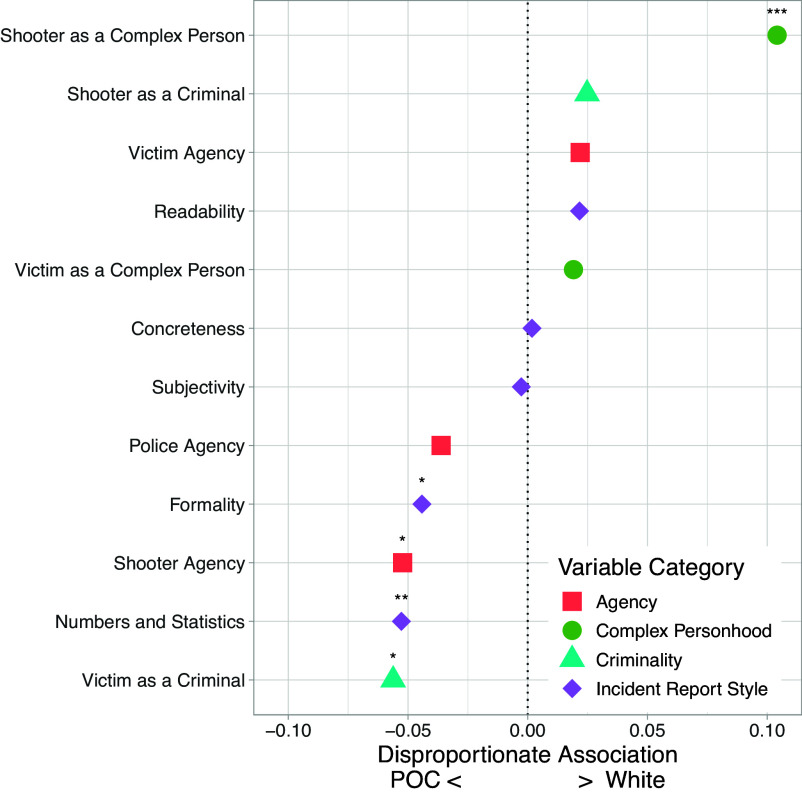
Disproportionate association of continuous linguistic features by neighborhood racial composition. Points are regression coefficients for each z-scored framing feature predicting race binary including controls. * is *P* < 0.05.

Our findings, shown in Fig. 3, show that articles about majority POC neighborhoods are more associated with increased formality and use of numbers and statistics, which are both more typically associated with an incident report style of writing, rather than a story. These features are also strongly associated with scope of the news source, with national news sources more likely to write articles like stories and local sources like incident reports (*SI Appendix*, Fig. S2).

In addition to stylistic differences, we see differences relating to how participants are framed based on their roles in the incident. Building on the findings from the previous section, we find that for incidents occurring in majority white neighborhoods, not only are shooters mentioned much more frequently in articles about majority white neighborhoods, but they are also framed as more complex people, while shooters in majority POC neighborhoods are portrayed with greater agency. The portrayals of victims are even more nuanced, as we see that although victims are more likely to be mentioned in majority POC neighborhoods, they are also framed as more criminal in those articles.

#### Ontology categories.

To aggregate these features into their larger conceptual categories in our ontology, we use the sum of all binarized constituent features to quantify the category (based on whether a content feature is present, or if the linguistic feature has a z-score above 0). We use each category in the ontology, as well as neighborhood and incident characteristics, as predictors in regressions with neighborhood racial composition as the dependent variable.

Our findings in Table 1 show that broadly, articles covering incidents in majority white neighborhoods focus more on the participants, framing them as more complex people, and more frequently mentioning or quoting people of authority. Articles covering incidents in majority POC neighborhoods tend to emphasize mortality and race while describing the incident in the form of a more objective report rather than a story and are also more likely to place the incident in the context of the broader narrative of gun violence in the United States.

**Fig. 4.**
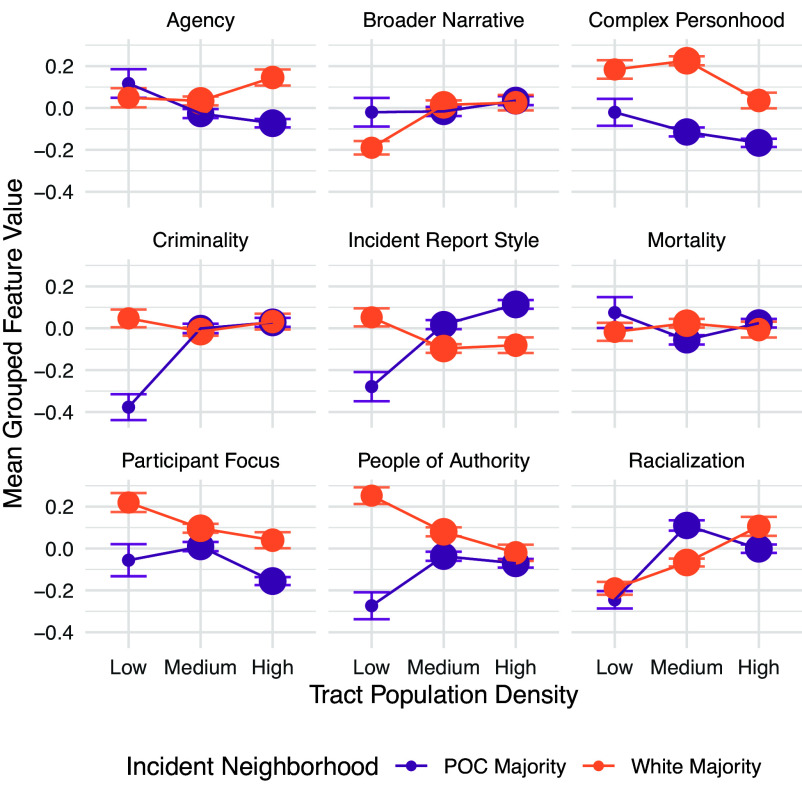
Grouped features by neighborhood racial composition and population density tercile. Points are group level means, and point size corresponds to the number of incidents in that group. Error bars represent 95% CIs.

We further use these larger conceptual categories to track trends across neighborhood population density, where we might expect differential associations since urbanness is known to be associated with increased rates of gun violence (32). As seen in Fig. 4, we find some direct trends: As tracts increase in density, we see that broader narratives and racialization increase while complex personhood and participant focus decrease. However, some associations are substantially modulated by race. Disparities in quoted people of authority are largest in low-density tracts, but are lessened in urban environments. Within low-density tracts, majority white neighborhoods show the strongest trend toward incident report style, while this trend reverses as density increases. Interestingly, racialization is highest for POC majority neighborhoods in the medium-density tercile, while white majority neighborhoods increase in racialization as density increases, suggesting a greater focus on race in relatively urban, white communities. Full regression outputs are provided in *SI Appendix*, Table S7.

## Discussion

Our findings offer a large-scale picture of the role of race in media depictions of gun violence in the United States. Across a national sample of articles linked to specific incidents of gun violence, we identify evidence for systematic disparities in coverage, content, and framing relative to the racial composition of the neighborhood in which incidents occurred, even when controlling for features of the incident and neighborhood.

The coverage differences we identify are stark and substantial: For critical categories like mass shootings and school shootings, incidents occurring in white neighborhoods receive roughly double the amount of coverage compared to those occurring in POC neighborhoods, and the reverse is true for officer-involved shootings. Existing work has established similar race-related disparities in coverage for particular cities or types of incidents (7, 8, 15, 33); we show these disparities are more generalized than previously documented, holding across the country and robust to incident-level controls.

To understand how the content of these representations might differ, we developed an ontology of broad linguistic categories relevant to media depictions of gun violence and a set of directly measurable, interpretable features that constitute them. Tracking these features across the dataset, our results first extend prior findings on specific aspects of representation. We confirm existing analyses showing a greater emphasis on the complex personhood of participants for incidents in white neighborhoods (19). An article for which all six of our complex personhood features appear has 2.5 times the odds of being from a white neighborhood compared to one in which none appear.

**Fig. 5.**
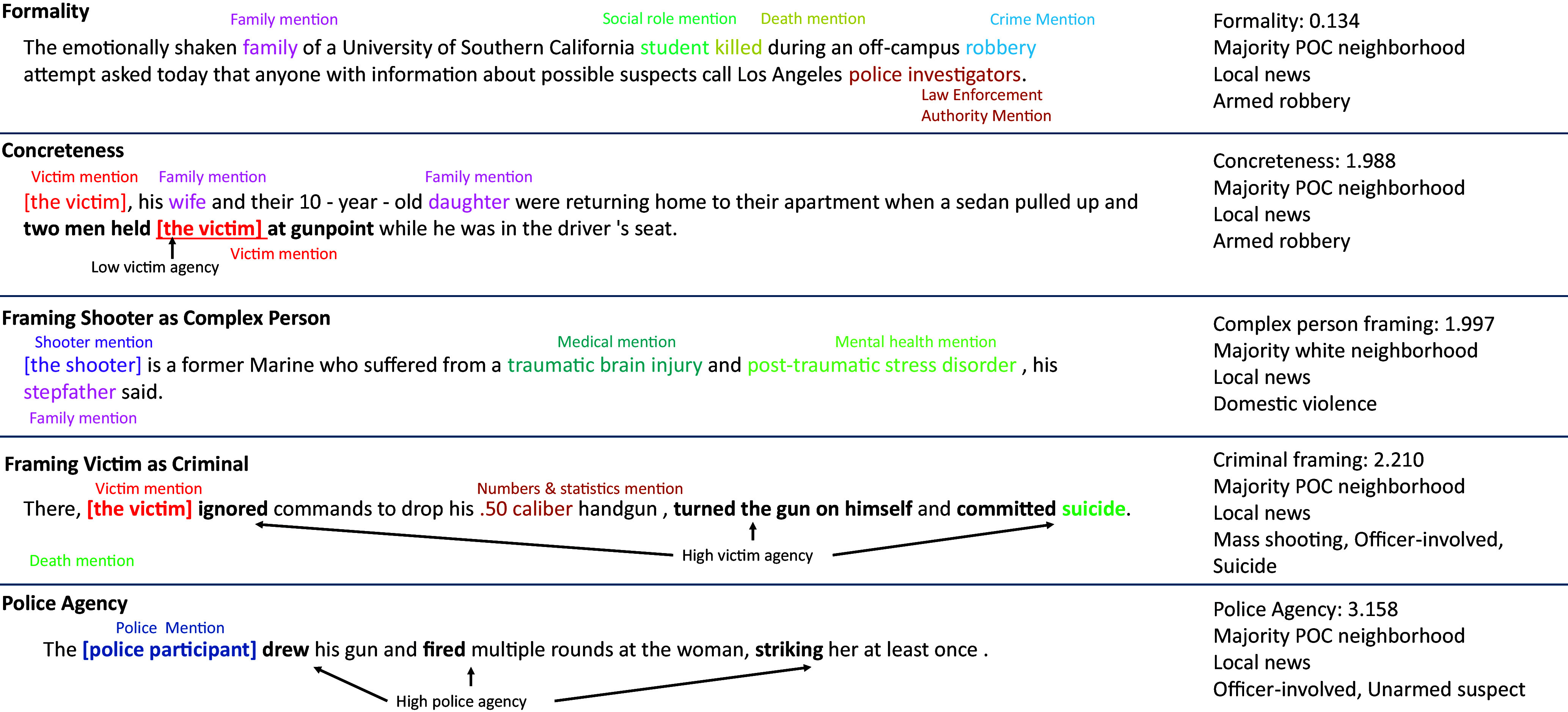
Example sentences labeled with features quantified in the analyses. Numeric values of features are z-scored, and reflect the score for the sentences shown, except for police agency, which is a score for the whole article where this sentence appears.

Our findings on implicit race add nuance to prior work showing strong disparities for particular types of incidents such as lone shooters (17). We replicate this in our raw data but we find that differences in baseline probability of appearance are largely explained by incident-level controls. Nevertheless, when implicit racial language appears, it is used more frequently for POC neighborhoods, averaging 0.79 additional mentions for a median-length article. In addition, we also find evidence of more subtle differences related to certain racialized stereotypes, with increased mentions of crime and framing of victims as criminals when an incident occurs in a majority POC neighborhood. (see Fig. 5 for examples of features).

Explicit race-related language also plays a role. We know from prior research that the US public is less likely to support gun control policies after reading an article about a Black victim of gun violence than a white victim (7); here we find that when race is mentioned in articles, 62% of the time these mentions exclusively reference people of color, while the same is only true 13% of the time for white people (*SI Appendix*, Fig. S8), suggesting a pattern of overexposure to news discussing POC in particular as involved in crime and violence (25).

Prior researchers have observed qualitatively and in case studies that depictions of the shooter and the attention of lawmakers or external parties are additional critical dimensions of media representation in this domain, particularly for mass shootings (8, 34). Our findings show that these disparities extend to representations of all types of gun violence: In white neighborhoods, articles tend to disproportionately discuss the shooter and factors relating to their complex personhood, both in terms of implicit framing and explicit mentions of mental health. Articles about majority white neighborhoods are also significantly more likely to include quotes from voices of authority such as police and local politicians, and this effect is accentuated in less urban environments. Our analyses cannot address whether this is due to increased effort at seeking out these contributions on the part of journalists, or an increased willingness to become involved on the part of the quoted authorities, but in either case, the result is that media representations depict greater engagement from those parties when the incidents occur in majority white areas.

In a recent qualitative study, victims of gun violence reported that harms from news coverage include being seen as a criminal or somehow responsible for the incident, feeling dehumanized or depersonalized, and coverage perpetuating fears about safety (3). Our evidence suggests that these harms are more likely to accumulate in communities of color, particularly decreased participant focus and complex personhood and increased incident report style and mortality.

The estimates of association from our feature-level models with controls in Figs. 2 and fig. 3, correspond to odds ratios ranging from 0.66 to 1.52. Though these could be described as small effect sizes for any particular linguistic feature (35), we argue that collectively these effects are evidence for a constellation of systematic differences in language associated with neighborhood race that we expect to meaningfully accumulate across the media landscape.

As a baseline measure of this, as mentioned at the onset of this article, we found that an LLM-based machine learning model in a balanced setting could achieve an accuracy of 75.9% at predicting neighborhood race. Fitting a logistic regression model solely on our interpretable features with leave-one-out cross-validation on the same article set as the aforementioned LLM, we obtain a generalization accuracy of 59.7%, suggesting first that our features explain a substantial portion of the representational disparity by neighborhood composition. However, the fact that LLMs capture yet more separability across articles by race also suggests that findings from our interpretable features represent a minimum bar for the level of disparity these features describe.

Future research is needed to better understand the causes and effects of these linguistic differences. This could include, for example, investigating the influence of journalist demographics on coverage patterns, how particular features may impact public perceptions, or whether newsroom interventions can be effective at mitigating disparate impacts in coverage. The methodological approach in this work is primarily descriptive, but the measurement tools released with this paper could be further used to identify or match stimuli for such future work.

Taken together, our findings provide evidence for large-scale disparate impact in media representations of gun violence. The results suggest an overall pattern of personalization in articles about majority white neighborhoods, where incidents are isolated events in which the participants and their social roles beyond the event are at stake, and authority figures are more engaged with the outcomes. By contrast, media representations in majority POC neighborhoods disproportionately emphasize crime and frame victims as criminal, and incidents are more frequently contextualized as part of the broader narrative of gun violence by referencing prior incidents, policy, or the issue of gun violence itself. Real-world gun violence disproportionately impacts communities of color, and our findings reveal that media coverage may compound these inequities through differential patterns of representation at scale.

## Materials and Methods

### Preprocessing and Matching.

Our dataset consists of 35,991 articles from US news sources between 2014 and 2023, which were sourced from the News on the Web (NOW) corpus, a large source of traditional news media articles posted online (26). Each article in the dataset covers a single incident of gun violence resulting in injury or death from the preceding week.

The incident information in our dataset comes from the Gun Violence Archive (GVA) (1). GVA collects data from over 7,500 sources, including police and government organizations as well as news reports, and manually annotates each incident, often including multiple sources of information, to ensure accuracy. Incident metadata includes features of the incidents, such as whether the incident was a school shooting or involved drugs, as well as available information on those involved and latitude, longitude, and street address where the incident occurred. Although data from GVA do not perfectly cover or represent all incidents of gun violence, it is a large-scale, public, and manually verified dataset that is available for the whole country and has been found to have excellent (99.0%) precision and good (81.1%) recall (36). Police department data can be more detailed and complete, but tends to vary widely city to city and is not aggregated nationally (12), and therefore GVA is the best source for our purposes. We used a subset of the GVA database, selecting only incidents within our timeframe (2014–September 1, 2023) where at least one person was injured or killed. Our dataset from GVA contained a set of 375,113 incidents; only 15,570 of those incidents remained after our rigorous matching and filtering process.

In order to be matched, articles must contain at least one primary match: a name or name variant of a participant, or the address where the incident took place; if the article contains only one primary match it must also contain at least one secondary match: the city, county, or place name where the incident occurred. We manually validated this matching algorithm and found it to have very high accuracy (further details on matching and validation in *SI Appendix*, section 1.C). The matched articles were then filtered so that the final dataset contained only articles that covered exactly one incident that occurred in the week before publication; that single incident was identified as the “primary incident” to differentiate from earlier incidents that articles might have also referenced. Using latitude and longitude of the location of the incident, the census tract where the primary incident took place is identified, and we used demographic data from the American Community Survey (ACS) to identify whether the location was located in a neighborhood that is majority white (≥60% non-Hispanic white population), majority POC (≤40% non-Hispanic white population), or mixed. The incidents occurring in mixed neighborhoods were omitted from this analysis but were included in analyses testing different definitions of race (see *SI Appendix*, section 6 for details).

Each of the top 600 most frequent news sources was manually identified for scope as “national” based on the following criteria inspired by ref. 37: 1) the source has no clear headquarters or area of reporting and 2) the source covers news in three or more states and/or include international coverage. Each article was then identified as coming from a national news source, or a local (non-national) news source.

Our final dataset of articles only covers a set of 15,570 unique incidents, mainly due to the fact that not all incidents in the GVA dataset had participants with publicly available names; because our matching system relied heavily on names to ensure high precision, any incident without names was significantly less likely to have a matched article. In addition, NOW does not scrape every article from every news source every day but rather collects a random sampling, which means coverage of some incidents may be missed. Furthermore, because our analysis relied on the neighborhood composition of an incident, we had to omit articles where many recent incidents are listed together. As a result, the set of incidents in our dataset does not include all incidents to ever get coverage, but rather describes the incidents that get their own articles.

### Content Features.

Explicit topic mentions were identified using keyword lists we curated, covering topics including mentions of family, police, and mental health. Besides simple keyword lists, some features required syntactic analysis using spaCy;^†^ for example, identifying speakers or race mentions required dependency matching to accurately tag the relevant features. In addition, we identified “notable mentions,” or mentions of high-coverage incidents, identified using the matching algorithm, from at least one month before the article was published.

### Framing Features.

Quantification of agency has been used in prior research on gun violence, police violence, and dehumanization (24, 38). The agency metric uses syntactic parsing to identify agent and patient roles as well as an agency lexicon (30) to identify the average agentivity of a type of participant (on a scale from −1 as never agentive to 1 as always agentive); articles that never mention a particular participant role as either an agent or patient do not have an agency score for that participant role.

Because victims of gun violence have expressed feeling dehumanized when the news described their incidents with impersonality and/or as part of an aggregate (3), we measure the frequency of numbers and statistics per sentence in each article, using the cardinal, percent, date, and time tags from spaCy named entity recognition. Concreteness quantifies the tangibility of words and has been used in analysis of gun violence headlines and images (39); greater concreteness likely indicates a greater emphasis on incident events, such as guns and injuries, while lower concreteness might indicate a greater emphasis on feelings or ideas. The concreteness is the average concreteness rating of all words in an article using the lexicon by ref. 40.

Readability has been used in media studies to quantify the difficulty level of the vocabulary and sentence structures; decreased readability is associated with a more expository style, rather than simpler narration (41). We use the Flesch-Kincaid reading ease metric (42) in order to quantify readability. Formality has been shown to be associated with perceived credibility (43), and the feature was calculated using an XLM-Roberta-based formality classifier trained on XFORMAL (44). Subjective language was included as a check for the norm of objectivity in news reporting, and the metric was the percent of words in the article that were identified as subjective using the subjectivity lexicon from ref. 45.

We measure participant framing based on the method used in ref. 31 to identify implicit dehumanization using large language models (LLMs), by quantifying the probability a mention of a participant (victim/shooter) could be substituted with one of a set of lexical items representing a target framing category. We identified a set of semantically similar keywords for the **criminal** frame. The complex person keywords were borrowed from the complex person words used in the content study, though slightly adapted by removing vague or group terms (such as “family” or “member”).

The masked data used for participant framing predictions were created by replacing each occurrence of a participant’s name (including name variants, e.g. “Mr. Smith” for “John Smith”) based on the participant’s role (victim, shooter, or police participant). Using the MODERNBERT-LARGE masked language model, the probability of each identified keyword was calculated for each mask, and the probability of the frame was the sum of its constituent keywords (see *SI Appendix*, Table S2 for keyword lists). The final value of a participant framing variable was the z-scored log of the average probability across all relevant masked occurrences in an article. Articles without sentences mentioning participants in such a way as to be scored had feature values of 0.

### Statistical Analysis.

For all regressions in our analyses, both coverage and article features, we controlled for incident characteristics from the GVA metadata, specifically whether the incident: got national coverage; was a hate crime, school shooting, mass shooting (4 or more casualties), suicide, or accident; involved children, gangs, drugs, police officers, assault weapons, domestic violence, or a home invasion; resulted in no deaths or 4 or more deaths; and whether the names of victims or shooters are known according to GVA. In addition to the binarized incident controls, the regressions with article features also have neighborhood controls: median income, population density of the census tract, and education (percentage of adult residents with at least a high school degree).

In order to calculate the associations between race and the article features, we used logistic regression models predicting the binarized race variable. The quantity of interest for content features in this calculation is whether or not they occur at any point in an article and are therefore operationalized as binary indicators. Framing features are gradient measures operationalized as continuous, z-scored variables.

For the measure of disproportionate mentions per sentence, we identify the difference in means between the two demographic groups for each content feature. Each content feature counts the number of times a keyword in that category appears, and in order to identify the focus or importance of a given feature when it does appear, we normalized by the number of sentences in the whole article, and z-scored that normalized value, omitting any articles where the feature does not occur at all. Using these feature values, we subtracted the mean value of articles about majority white neighborhoods from the mean value of articles about majority POC neighborhoods to determine the disproportionate mentions per sentence.

To ensure that our findings are robust, we tested analysis using different groupings for race (*SI Appendix*, Figs. S2–S4) and found consistently similar results. We also tested each feature as an independent hypothesis predicted by incident features and race to better understand how much of the variation in those features is accounted for by race; again, we find similar results (*SI Appendix*, Fig. S7), though Criminality becomes strongly associated with majority POC neighborhoods and Participant Focus and Agency lose significance (more details in *SI Appendix*, section 7).

### Machine Learning Predictions.

In order to test the magnitude of the raw difference between articles in majority white and POC neighborhoods, we use LLMs in a classification task. We first create a balanced dataset of 16,086 articles by pairing majority POC and majority white articles on incident characteristics, masking named entities and explicit mentions of race, and trimming each article to 1,000 tokens. We then train language models on that dataset to predict race from the text and find an average accuracy of 75.9% with an LLM-based classifier and 71.2% with a traditional SVM machine learning classifier, substantiating our findings that there are noticeable differences in articles about gun violence incidents in majority white and majority POC neighborhoods (full results in *SI Appendix*, section 4).

## Supplementary Material

Appendix 01 (PDF)

## Data Availability

Dataframes and scripts sufficient to replicate the findings described in the paper have been deposited in our publicly accessible GitHub repository and are available for download at https://github.com/rbagley/gunviolence (46). Due to licensing restrictions, we are not able to distribute raw news article data from NOW or incident metadata from GVA. For any use beyond replication, we provide scripts to replicate our full preprocessing, matching, and analysis pipeline for users who obtain these datasets independently from GVA at https://www.gunviolencearchive.org/ (1) and from NOW at https://www.english-corpora.org/now/.

## Footnotes

^*^https://www.gunviolencearchive.org/.

^†^https://spacy.io/.
