# Supplementary material for: Quantifying racial disparities in media representations of gun violence at scale
Source: Proc Natl Acad Sci U S A. 2026 Jan 16;123(3):e2505499123. doi: 10.1073/pnas.2505499123 (PMC12818404; doi:10.1073/pnas.2505499123)
Supplement: Supplementary file 1 — Appendix 01 (PDF) [file pnas.2505499123.sapp.pdf]

Supporting Information for

Quantifying Racial Disparities in Media Representations of Gun Violence at Scale

Ruth Bagley, Susan Burtner, Andrew Papachristos, and Rob Voigt

Ruth Bagley.
E-mail: ruth.bagley@northwestern.edu

This PDF file includes:

Supporting text
Figs. S1 to S9
Tables S1 to S9
SI References

Supporting Information Text

1. Dataset Curation

A. Incident Data. The incident metadata used in our dataset came from the Gun Violence Archive (GVA), which aims to manually document all incidents of gun violence that occur within the US since it began in 2013, with the recall steadily improving over time (1). For each incident, they have recorded significant metadata including available data about the participants, and whether the incident was officer-involved, occurred at a school, involved gangs, etc.

With their cooperation, we obtained their full dataset of incidents from their inception in 2013 to the present. While GVA documents all incidents involving guns (such as incidents where illegal guns were found but not used), we look specifically at the subset of incidents involving gun violence where at least one person was injured or killed.

To ascertain the racial makeup of the immediate area around where the incident occurred, we found which census tract the incident occurred in using the latitude and longitude of the incident given in GVA, based on the street address where the incident took place. We then linked the demographic information of the census tract to the incident, including median income, racial composition, population, and education (percent of adult residents with at least a high school degree). These variables were then used to identify the proportion of the population in the census tract that identifies as non-Hispanic and White (and no other race). These variables were gathered using the American Community Survey (ACS) 5-year dataset from the year of the incident (e.g. 2016-2020 for incidents in 2020), or the most recent year available if the incident is more recent than available census data.

B. Text Data. The source for our articles is the News on the Web (NOW) text dataset. News on the Web is an ongoing scrape of news articles since 2010; this dataset collects more than 300,000 articles per month from several thousand different sources including local and national news media outlets from the US and around the world. Our top ten sources with the most article matches are: New York Post, Fox News, Chicago Sun Times, New York Daily News, AL.com, Chicago Tribune, CBS News, SFGate, Washington Post, and ABC.

Articles in NOW come pre-tokenized (with spaces between every word and punctuation mark) and we use this tokenization in analyses to follow. We also note that for copyright purposes 5% of tokens are masked out in the dataset (ten out of every 200 words), but since this masking is randomly distributed we expect it will not interfere with any analyses we perform.

We subset this dataset to only articles appearing in US-based media sources within 2014-2023, and then use an initial keyword search to find any articles relating to gun violence. The keywords used in this search are: *gun(s), firearm(s), shot, shooting(s), murder(ed), gunfire, gunshot, assault rifle(s)*, and *assault weapon(s).*

This initial filter is very broad, and many of the articles that meet the criteria will not be depictions of particular incidents (as we aim to focus on) but rather incidental mentions of these keywords; this search is intended to be a first filter to provide a pool of candidate articles to be matched to gun violence incident metadata from GVA.

C. Data Linkage. To link the article and incident data, we compared each news text to all gun violence incidents from the previous 365 days and allowed for matches on the following primary characteristics: (1) participant names (and name variations) for both shooters and victims, (2) incident street addresses, and (3) source URLs.

Name variations are derived using regular expressions, allowing for different versions of the name in the GVA metadata. For example, a variant of 'John D. Smith' would be 'John Smith'. The variants used in the matching stage include the inclusion or exclusion of middle initials, suffixes, and titles.

These potential matches were verified with other features, such as city, state, or place name (such as if the incident occurred at a business or school). For the source URL matching, a perfect URL match was sufficient to merit a match. For the text-based matching, the article needed at least one primary match (name or address), and either another primary match (not including variants of the same participant's name) or a secondary match (city, state, or place name).

There are a variety of articles that discuss the social problem of gun violence more broadly; for our analysis we filtered the matched articles to only include those mentioning precisely one incident that occurred within a week of the article's publication. This filtering allows us to look at media depictions in the immediate aftermath of an incident; depictions discussing recent events in the context of less recent events are included in the dataset, but articles exclusively discussing older incidents are not included.

Because our matching algorithm necessarily relies heavily on names for matching, one important consideration is which incidents in the full GVA dataset include names. Names might be excluded by choice (if a victim does not want their name published) or lack of knowledge (such as if the shooter has not been identified). Another consideration is that much of the data in GVA is gathered from diverse sources - not only media reports, but also social media reports, obituaries, funeral home listings, and so on; if these reports lack this metadata, then we will simply not have access to it for a given incident. Descriptive statistics on the number of articles matched to a given incident in our final dataset are shown in Table S1.

C.1. Matching Algorithm Validation. In order to ensure the complex matching algorithm is effective, 50 matched articles were randomly selected and the matches were checked by hand. Of this subset, all but one were accurate matches, suggesting the precision is approximately 98%.

Because the GVA dataset contains over 370,000 incidents and we only matched articles to about 15,000 of those, we also checked whether the algorithm was missing a substantial number of matches. We randomly selected 50 incidents in the dataset, and found only one was matched to an article by our algorithm. Only 40% of these incidents had any named participants (a key

element that would allow matching), and none of the names occurred anywhere in the NOW dataset during the month when the incident occurred, suggesting NOW did not have articles about those incidents that our algorithm could have found but missed. The one matched incident did have a named participant, but was matched based on its URL, because the participant was not named in the article.

This validation suggests that the algorithm is effective at correctly matching incidents and articles without systematically missing large numbers of potential matches.

2. Content Mentions Methodology

Each feature in the content mentions was quantified using the criteria in Table S2, and then the feature was binarized for the statistical analysis to indicate if it ever occurred within the article. All quoted speakers were identified using syntactic dependency matching, based around the following verbs: say, state, add, identify, call, tell, observe, describe, report, mention, remark, discuss, acknowledge, demonstrate, support, clarify, and recognize. In order to extract the most information about the speaker, noun phrases that performed those verbs were extracted according to several different patterns, including full names, a single name (e.g. a last name), names including a title, names of people identified as part of a group (e.g. "Officer Johnson of the Chicago Police Department"), or names with appositives (e.g. "Mary, the victim's mother"). These extracted phrases were then matched to the keywords found in Table S2 to identify who was quoted.

A. Participants. For each of the features relating to participants in both content and linguistic features (e.g. shooter names, victim agency), mentions of the participants by name first had to be identified. Because participants are not always mentioned by their full name throughout the article, a broader set of name variants was generated for each participant than in the initial phase of matching incidents to articles. The expanded name variants not only included names excluding and including titles, suffixes, and middle initials, but also combinations of title with last name (e.g. 'Mr. Smith' for 'John Smith'), or 'Smith' on its own. In addition, some participants are identified with nicknames in GVA, so "John 'Johnny' Smith" would also match to both "John Smith" and "Johnny Smith". These name variants were then matched to any named entities in the text identified by spaCy to facilitate more accurate matching.

The three features measuring participant mentions are **Police Participants**, **Shooter Names**, and **Victim Namess**. For each type of participant (civilian shooter, civilian victim, and police participant), mentions of them by name (including name variants) are counted within the context of the article.

3. Linguistic Features Methodology

A. Participant Features. Agency was quantified by identifying all phrases where a type of participant (civilian victim, civilian shooter, or police officer) was either a subject or object, determining if it was a high agency phrase based on the verb involved and the participant's role as subject/object according to the Connotation Frames of Power and Agency lexicon (2) and calculating the proportion of those phrases where the participant has high agency.

B. Implicit Framing. The implicit framing features (**Shooter as Criminal, Victim as Criminal, Shooter as Complex Person, and Victim as Complex Person** were quantified following the process laid out in (3). First, keywords were identified relating to 'criminal' and 'complex person'. The keywords for complex person were the set of words used in the 'Social Role' and 'Family' content mentions (see Table S2). However, the 'criminal' category was new, and therefore we selected several related words to fill out the category, specifically: *criminal, felon, fugitive, convict, thug, gangster, terrorist.*

Sentences that mentioned participants by name were identified, the names were masked, and the sentences were divided into participant type: civilian victim, civilian shooter, and police participant. Each of those participant types then separately went through the prediction process. First, sentences that do not mention that kind of participant by name exactly once are filtered out. For the remaining sentences, mentions of the participants are replaced with a mask token, and using a ModernBERT-base masked language model*, the probability of that mask token being any word in the categories described above was predicted. These probabilities were summed across category to create a total probability of the name being replaced with a word in the Criminal, or Complex Person categories. A total of about 131,000 sentences had those probabilities quantified in this process.

We validated these features by randomly selecting 25 pairs of example sentences from the top 20% and the bottom 20% for each feature, and one author manually annotated which of the pair should have the higher score. The accuracy for Victim as Criminal was 68%, Victim as Complex Person was 76%, Shooter as Criminal was 72%, and Shooter as Complex Person was 72%. These features are identifying subtle patterns and therefore the scores are not perfect, but they are substantially better than chance. We had also tested 'Police as Hero', to identify whether police officers involved in incidents of gun violence were framed differently, but that validation for that feature was only 56%, and therefore it was omitted from the final analysis.

C. Stylistic Features. In order to measure **formality**, we first used spaCy to identify all sentences in each article that contained both a verb and a noun/pronoun/proper noun; because of the random masking in the NOW dataset, sentences could be cut very short, which would likely result in less accurate formality measures. The xlmr_formality_classifier from huggingface† was then used to quantify the formality of each sentence, and those scores were averaged across each article to create a formality metric for the article as a whole.

* https://huggingface.co/answerdotai/ModernBERT-base
† https://huggingface.co/s-nlp/xlmr_formality_classifier

Subjectivity was measured by identifying the subjectivity and polarity of each non-punctuation token in an article using the subjectivity lexicon by (4). Each word in the lexicon is associated with high or low subjectivity and positive/negative/neutral polarity, and also specifies if there is a need for lemmatizing or identifying the part of speech. The subjective words score for a whole article is the proportion of non-punctuation tokens that are identified as high subjectivity (of any polarity).

The **readability** metric used spaCy readability[‡] to quantify the Flesch-Kincaid reading ease score (5) for each sentence in an article. These sentences had the names masked (e.g. 'shooter_name was arrested after the incident.') and, as with formality, were filtered for length and part of speech, and the sentence scores were averaged to quantify the readability of the article as a whole.

In order to measure the **concreteness** of an article, we used the concreteness lexicon (6). First, the concreteness rating of each token in the article was identified using the word and its part of speech (tokens not in the lexicon, such as punctuation, were ignored). Then the concreteness scores were averaged across the article to form the final concreteness metric of the article.

The frequency of **numbers and statistics** was measured by using spacy to count the number of named entity phrases identified as DATE, CARDINAL, PERCENT, or TIME. This sum was then normalized by the number of sentences in the article to account for variation in article length.

4. Feature statistics

For all of the content mentions and linguistic features, we include descriptive statistics in tables S3 and S4. For the linguistic features, we include the mean feature value for the full dataset and for each of the neighborhood composition groups, along with the proportion of articles in each neighborhood group that have a value greater than the overall median. As a note, some features have clusters at the median, and therefore the proportion of articles above that value is less than 50%. . For the content mentions features, we include the mean feature value for all articles where the feature appears, along with the proportion of articles that mention a given feature at all. We also include the prevalence ratio or how much more likely it would be for a feature to appear in an article about a POC neighborhood than in a majority white neighborhood; in this statistical metric, values greater than 1 signify that the feature is more likely to occur for the POC neighborhoods. For instance, the prevalence ratio for 'Mental Health' is 0.67, meaning that an article about an incident in a majority POC neighborhood is 33% less likely to include any mention of mental health than one about a majority white neighborhood.

5. Scope as Outcome

In addition to predicting the racial composition of the neighborhoods, we also ran regressions that looked at the scope of the news source that published the article, in order to identify any trends that might be present there. To do this, we used the same regression structures as for race, but instead predicted whether the source was local or national. We plot the regression coefficients for scope and race in Figures S1 and S2. We find that the scope of the news source does play a role in these portrayals: national news sources are more likely to write about the incident with more subjective language, portray participants with more agency, and emphasize police participants, while local news sources are more likely to use formal, concrete language with more frequent numbers and statistics. Local and national news sources also treat victims differently: local news sources mention victims more frequently and frame them as complex people, while national news sources are more likely to frame victims as agentive and criminal. Taken together, this suggests that national news sources tend to write articles that more closely resemble stories, while articles in local news sources are framed more like incident reports.

6. Language Model Predictions

In order to predict racial demographics of articles using language models, we first created a balanced dataset of 16,086 articles by pairing majority POC and majority white articles on incident characteristics. We then used spaCy's named entity recognition to mask any tokens identified as PERSON, GPE, ORG, LOC, and NORP. We then used four different models for comparison, all using 5-fold cross-validation: a unigram/bigram linearSVC model and three large language models (DeBERTa-base[§], ModernBERT-base[¶], and ModernBERT-large[‖]) to predict whether an article was written about a majority white or majority POC neighborhood based on the masked article text. Results are in Table S5.

As a robustness check, we identified the most predictive words according to the SVM, and none of the tokens were obviously related to race. Specifically, *gunfire, block, child, he was, avenue, to be, with gun, told [ORG], sheriff department, mayor* were most associated with majority POC neighborhoods, and *parents, they believe, downtown, closed, student, and, in [GPE], coy, the capitol, capitol* were most associated with majority white neighborhoods.

7. Coverage Full Results

As a robustness check for how coverage is quantified, we considered three different variables for coverage: the number of articles that cover an incident, the length of the articles, and whether the incident gets covered by a national source. Table S6 shows the results of four different models, each predicting one metric for coverage, and one model predicting a binarized value for

[‡] https://spacy.io/universe/project/spacy_readability
[§] https://huggingface.co/microsoft/deberta-base
[¶] https://huggingface.co/answerdotai/ModernBERT-base
[‖] https://huggingface.co/answerdotai/ModernBERT-large

article count (whether the incident got more than one article). All four coverage models have roughly similar results, and therefore article count (or the number of articles matched to the incident) was the only coverage metric used in the main body of the paper.

In addition, we tested whether other neighborhood features played a substantial role in coverage. Table S7 shows how well the neighborhood demographic features predict coverage on their own, and Table S8 shows the results of regression models that use both incident and a broader set of neighborhood features based on the concentrated disadvantage index (7).

8. Definition of Race

In the main paper we used a 60% supermajority definition of race for the two groups, where the groupings were determined based on whether the incident occurred in census tracts with at least 60% non-Hispanic white residents (white), or at most 40% non-Hispanic white residents (POC), and incidents occurring in the most mixed areas were omitted from the analysis. Ultimately the operationalization of neighborhood race is a decision related to study design.

For robustness, we also considered other definitions of race, used regressions predicting race from the ontological categories as a point of comparison. The results using our model in the main paper are in Fig. S3, which can be compared to the other four definitions of race: simple majority (Fig. S4), three-way split (Fig. S5), continuous scale (Fig. S6), and explicit mentions (Fig. S7).

A. Simple Majority. A simple majority split grouped the data into white and POC groups based on whether the census tract had at least 50% non-Hispanic white residents. This split allowed for more data to be included in the analysis (43,864 articles rather than 35,991), but might not accurately convey differences between white and POC neighborhoods if the mixed neighborhoods are split into both groups. Under this definition we find similar results to the supermajority operationalization. The results using this definition of race are found in Figure S4.

B. Three-way Split: Majority White, Mixed, and Majority POC. In order to test whether our supermajority split is reasonable, we also do a comparison of the two majority groups with the middle "Mixed" group that is dropped from the dataset in the main analyses. The results comparing regressions predicting Majority White vs. Mixed and Majority White vs. Majority POC (the findings in the main paper) are in Fig. S5. We can see that in some ways the Mixed group aligns with the Majority POC group (Mortality, Participant Focus, and Incident Report Style), others align more with Majority White (e.g. Agency, People of Authority), or lie somewhere in the middle (e.g. Broader Narrative, Racialization). This suggests that the Mixed group is distinct from the other two groups, rather than a group existing midway along a continuum between the majority groups, and conflating them might paint over some nuances.

C. Continuous Scale. An additional robustness check for our race definition is by using a linear regression to predict the percent of the neighborhood that identify as white. The findings, shown in Fig. S6, are very similar to the definition of race we use in the main body, suggesting that the findings are robust. However, this definition produces slightly less interpretable results, and therefore we chose to use the neighborhood supermajority groups.

D. Race Mentions. Instead of using neighborhood race, we also considered using the race of the participants, which might capture other nuances (e.g. coverage of a Black vs. white victim in a majority white neighborhood). Because GVA does not collect information on individual race, we identified explicit mentions of the race of people within the articles. However, not many articles actually mention race explicitly, and when they do, in the vast majority of cases, it is mentions of non-white races (e.g. Black or Asian). If a participant is described as white, it is usually only when another race is also mentioned, as seen in Fig. S8. Consequently, racialization is more likely when someone is described as white, as seen in Fig. S7. Regressions using this definition of race must use a much smaller and less balanced dataset, and prior works have used neighborhood-level features in studies of gun violence (e.g. (8, 9)), and therefore we chose to use neighborhood demographics as a proxy for race.

9. Alternative Matching-Based Estimation Strategy

Although this is not a causal study, we were interested in quantifying the relationship between neighborhood racial composition and article features. We know that race cannot be fully captured by a single metric and is better understood as a collection of many elements (10), yet we are using a single race variable in our analyses. As a robustness check of our findings, we test each article feature as an independent hypothesis using coarsened exact matching (CEM) (traditionally a causal inference method) to see how much race accounts for the variation in the article features, controlling for incident characteristics and balancing on neighborhood characteristics.

These analyses used CEM to weight the regressions so that the two race groups were balanced based on median income, population density, and education (percent of the adult population with at least a high school degree). These characteristics, drawn from census data, were "coarsened" by dividing them into 3-5 quantiles, and the coarsened values were matched. For the actual regressions, a linear or logistic regression was run for each article feature, where the independent variables were incident features and neighborhood race, predicting a single outcome article feature. The metric for the strength of the relationship was the coefficient for the race variable. Results from this analysis (shown in Fig. S9), are broadly similar, though not identical, to the main regressions. For instance, Broader Narrative, Racialization, and Incident Report Style remain strongly associated with majority POC neighborhoods, while Complex Personhood and People of Authority are strongly associated with majority

white neighborhoods. However, Agency, Participant Focus, and Mortality lose significance, and Criminality becomes strongly associated with majority POC neighborhoods, which is not present in the main analyses.

10. Population Density Analyses

In the paper we aim to add further neighborhood-level nuance by explicitly testing for relationships between neighborhood racial composition, population density, and the nine grouped categories in our linguistic ontology. We implement this by dividing all tracts in the dataset into terciles (“Low”, “Medium”, and “High”) and fitting a regression testing for significant interaction effects between these density terciles and each of the nine ontology categories.

The full regression results are shown in Table S9 and discussed in the main body of the paper.

Table S1. Distribution of the number of articles matched to incidents in our dataset

Count	15570
Mean	2.456
Min	1
25%	1
50%	1
75%	1
Max	669
Std. Dev.	11.194

Table S2. Content features and how they were identified or quantified

Feature name	Method	Keywords/Matching Criteria
Race	Syntactic parsing and keywords	white, black, asian, hispanic, latin, african, caucasian, latino, latina] modifying [prisoner, teen, worker, member, kid, lawyer, victim, officer, wife, american, neighbor, male, population, person, cop, woman, family, boy, girl, mother, daughter, son, father, man, suspect, resident, teenager, parent, child, leader, female, people, women, men, families, children]
Implicit Race	Keywords only	thug, thuggery, thugs, gang, gangs, gangsters, gangster, gangbanger, gangbangers
Bias	Keywords only	racism, racist, bias, biased, supremacy, supremacist
Policy	Keywords and parts of speech	permit (n), amendment, legislation, open carry, background check, concealed carry, gun control, gun law, gun rights, waiting period, red flag, proposed bill, policy
Gun Violence	Keywords only	gun violence
Notable Incidents	Incident matching	Using initial incident matching criteria, any mentions of top 5% most covered incidents by year that occurred greater than one month before the article was published
Legal	Keywords only	criminal, judge, lawyer, attorney, bailiff, court, courthouse, lawsuit, bail, appeal, arraignment, accused, warrant, felony, conviction, evidence, defendant, jury, parole, parolee, plaintiff, prosecutor, witness, public defender, guilty
Crime	Keywords only	crime, arrest, handcuff, crime scene, scene of the crime
Death	Keywords only	succumb, suicide, murder, manslaughter, slaughter, bloodshed, coroner, autopsy, funeral, grief, grieve, mourn, bereave, coffin, kill, killer, bury, fatal, dead, execution, death, morgue, die, homicide, demise, massacre, corpse, deceased, lethal, casualty, doa, passing, flatline, heart stopped, pass away, pronounce dead, life lost, lose life, lose heartbeat
Medical	Keywords only	injury, injure, wound, ambulance, emt, medical, medicine, hospital, blood, bleed, heartbeat, surgery, icu, paramedic, paralysis, paralyze, concussion, tbi, stretcher, bandage, body bag, life support, critical condition, intensive care, operating room, emergency room
Family	Keywords only	cousin, aunt, uncle, mother, father, mom, dad, stepmom, stepdad, stepmother, stepfather, sister, stepsister, brother, stepbrother, son, stepson, daughter, stepdaughter, grandmother, grandfather, grandma, grandpa, niece, nephew, sibling, parent, grandparent, child, family, wife, husband, boyfriend, girlfriend, fiancee
Social Role	Keywords only	friend, neighbor, boss, employee, student, teacher, resident, member, coach, teammate, team, mentor, leader, volunteer
Mental Health	Keywords only	schizophrenia, schizophrenic, depression, depressed, anxiety, autism, autistic, psychopath, sociopath, suicide, suicidal, psychiatric, psychological, ptsd, bipolar, asd, bpd, psychotic, psychosis, alcoholic, alcoholism, ocd, mental health, mental illness, mental hospital, mentally ill
Law Enforcement Authority	Keywords only	sheriff, deputy, detective, captain, lieutenant, investigator
Officers	Keywords only	officer, policeman, policewoman, cop, patrol, trooper, corporal
Quoted Police	Keywords and syntax	police, officer, sheriff, deputy, cop, trooper, policeman, detective, sergeant, enforcement, lieutenant, investigator, patrol, capt, sgt, lt
Quoted Local Politician	Keywords and syntax	Titles including 'mayor' or 'council', or the names of mayors in the biggest 10 cities in the US: Bill de Blasio, Eric Garcetti, Lori Lightfoot, Sylvester Turner, Kate Gallego, Jim Kenney, Ron Nirenberg, Todd Gloria, Eric Johnson, Sam Liccardo, Steve Adler, Mike Duggan, Eric Adams, Karen Bass, Brandon Johnson
Quoted National Politician	Keywords and syntax	Names including: senator, representative, sen, president, pres, US, United States, U.S., White House, senate, governor OR Names of presidents, vice-presidents, majority/minority leaders: Biden, Kamala Harris, Trump, Pence, McConnell, Schumer, Scalise, Jeffries, Pelosi
Quoted Personal Relation	Keywords and syntax	Last name is the same as a participant, or described using any of: cousin, aunt, uncle, mother, father, mom, dad, stepmom, stepdad, stepmother, stepfather, sister, stepsister, brother, stepbrother, son, stepson, daughter, stepdaughter, grandmother, grandfather, grandma, grandpa, niece, nephew, sibling, parent, grandparent, child, family, wife, husband, boyfriend, girlfriend, fiancee, friend, neighbor, colleague, coworker, boss, employer, employee, manager, relative

Table S3. Distribution statistics for linguistic features, including the mean feature values (with standard deviation) and proportion of articles above the median value for the full dataset, as well as the subsets of articles in majority white and majority POC neighborhoods. We also include relative risk (with confidence interval) as a statistical measure of how much more likely an article about a POC neighborhood is to have an above average value for a given feature than an article about a majority white neighborhood.

	Mean Feature Value			% of Articles Above Median		
Feature	Overall	White	POC	White	POC	Prevalence Ratio for POC neighborhoods
Concreteness	2.63 (0.12)	2.62 (0.11)	2.64 (0.12)	46.62	52.54	1.13 [1.1, 1.15]
Formality	0.95 (0.06)	0.95 (0.06)	0.95 (0.06)	49.13	50.65	1.03 [1.01, 1.05]
Subjectivity	0.02 (0.01)	0.02 (0.01)	0.02 (0.01)	51.69	48.54	0.94 [0.92, 0.96]
Police Agency	0.47 (0.46)	0.46 (0.47)	0.47 (0.45)	48.55	50.91	1.09 [1.05, 1.13]
Shooter Agency	0.34 (0.4)	0.35 (0.38)	0.34 (0.42)	52.07	48.12	0.8 [0.77, 0.82]
Victim Agency	0.21 (0.3)	0.22 (0.31)	0.21 (0.3)	49.34	45.96	0.93 [0.9, 0.96]
Victim as a Criminal	-6.88 (1.18)	-6.98 (1.23)	-6.8 (1.13)	46.31	52.79	1.13 [1.09, 1.18]
Victim as a Complex Person	-3.72 (1.11)	-3.7 (1.11)	-3.74 (1.1)	51.01	49.23	0.96 [0.92, 0.99]
Shooter as a Criminal	-5.91 (1.08)	-5.93 (1.1)	-5.89 (1.07)	49.51	50.52	0.72 [0.69, 0.74]
Shooter as a Complex Person	-4.03 (0.98)	-3.94 (0.98)	-4.11 (0.98)	53.33	46.44	0.61 [0.59, 0.64]
Readability	65.24 (9.73)	64.72 (9.27)	65.62 (10.05)	47.75	51.69	1.08 [1.06, 1.11]
Numbers and Statistics	0.64 (0.31)	0.62 (0.28)	0.66 (0.33)	48.08	51.44	1.07 [1.05, 1.09]

Table S4. Distribution statistics for content mention features, including the mean feature values (with standard deviation) and proportion of articles above the median value for the full dataset, as well as the subsets of articles in majority white and majority POC neighborhoods. We also include relative risk [with 95% confidence bounds] as a statistical measure of how much more likely an article about a POC neighborhood is to have each feature than an article about a majority white neighborhood.

	Mean Frequency per Sentence			% of Articles with Feature			
Feature	Overall	White	POC	Overall	White	POC	Prevalence Ratio for POC neighborhoods
Social Role	0.84 (0.78)	0.92 (0.84)	0.77 (0.71)	46.50	52.39	42.06	0.8 [0.79, 0.82]
Family	1.67 (1.56)	1.67 (1.58)	1.68 (1.54)	55.03	59.71	51.50	0.86 [0.85, 0.88]
Law Enforcement Authority	0.5 (0.25)	0.52 (0.27)	0.47 (0.24)	47.48	53.21	43.17	0.81 [0.79, 0.83]
Legal	0.75 (0.53)	0.76 (0.53)	0.74 (0.53)	59.06	62.24	56.66	0.91 [0.89, 0.93]
Mental Health	0.53 (0.59)	0.56 (0.62)	0.51 (0.56)	7.58	9.36	6.24	0.67 [0.62, 0.72]
Officers	0.4 (0.16)	0.39 (0.17)	0.4 (0.16)	55.99	54.33	57.25	1.05 [1.03, 1.07]
Crime	0.42 (0.18)	0.41 (0.17)	0.43 (0.19)	53.14	51.44	54.42	1.06 [1.04, 1.08]
Medical	0.92 (0.73)	0.89 (0.73)	0.94 (0.74)	68.50	68.69	68.36	1.0 [0.98, 1.01]
Death	1.59 (1.11)	1.6 (1.13)	1.58 (1.09)	87.05	87.84	86.45	0.98 [0.98, 0.99]
Race	0.46 (0.31)	0.45 (0.33)	0.47 (0.3)	10.22	7.63	12.16	1.59 [1.49, 1.7]
Bias	0.51 (0.5)	0.5 (0.45)	0.52 (0.54)	4.76	4.69	4.80	1.02 [0.93, 1.12]
Policy	0.55 (0.61)	0.57 (0.72)	0.53 (0.5)	10.67	11.40	10.12	0.89 [0.84, 0.94]
Notable incidents	0.33 (0.18)	0.32 (0.19)	0.34 (0.17)	4.25	3.37	4.92	1.46 [1.31, 1.62]
Quoted National Politician	0.35 (0.18)	0.36 (0.18)	0.35 (0.18)	5.19	5.99	4.60	0.77 [0.7, 0.84]
Quoted Local Politician	0.39 (0.18)	0.39 (0.18)	0.39 (0.18)	24.89	25.43	24.49	0.96 [0.93, 1.0]
Quoted Police	0.42 (0.2)	0.43 (0.21)	0.42 (0.2)	27.88	30.97	25.56	0.83 [0.8, 0.85]
Quoted Personal Relation	0.4 (0.2)	0.41 (0.21)	0.39 (0.19)	15.42	17.42	13.92	0.8 [0.76, 0.84]
Gun Violence	0.47 (0.4)	0.46 (0.39)	0.47 (0.41)	5.40	4.42	6.14	1.39 [1.27, 1.52]
Implicit Race	0.33 (0.13)	0.31 (0.11)	0.34 (0.13)	4.89	3.45	5.98	1.74 [1.57, 1.92]
Shooter Names	1.41 (1.15)	1.44 (1.2)	1.37 (1.1)	50.55	61.02	42.67	0.7 [0.69, 0.71]
Victim Names	1.3 (1.1)	1.33 (1.11)	1.28 (1.09)	51.82	52.87	51.03	0.97 [0.95, 0.98]
Police Participants	1.3 (1.23)	1.25 (1.2)	1.33 (1.24)	13.79	12.49	14.77	1.18 [1.12, 1.25]

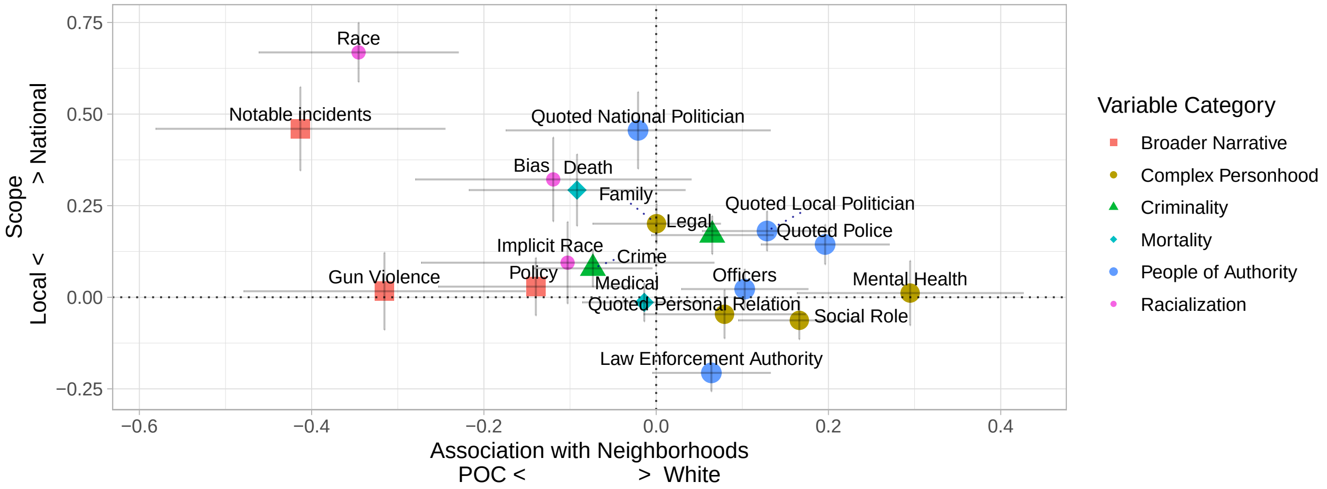

Fig. S1. Disproportionate mentions of content features by neighborhood racial composition and news source scope. Points are regression coefficients for each binarized content feature in regressions predicting race binary and source scope, and including controls. Color and shape are used to represent the variable's category in the ontology. Error bars represent 95% confidence intervals.

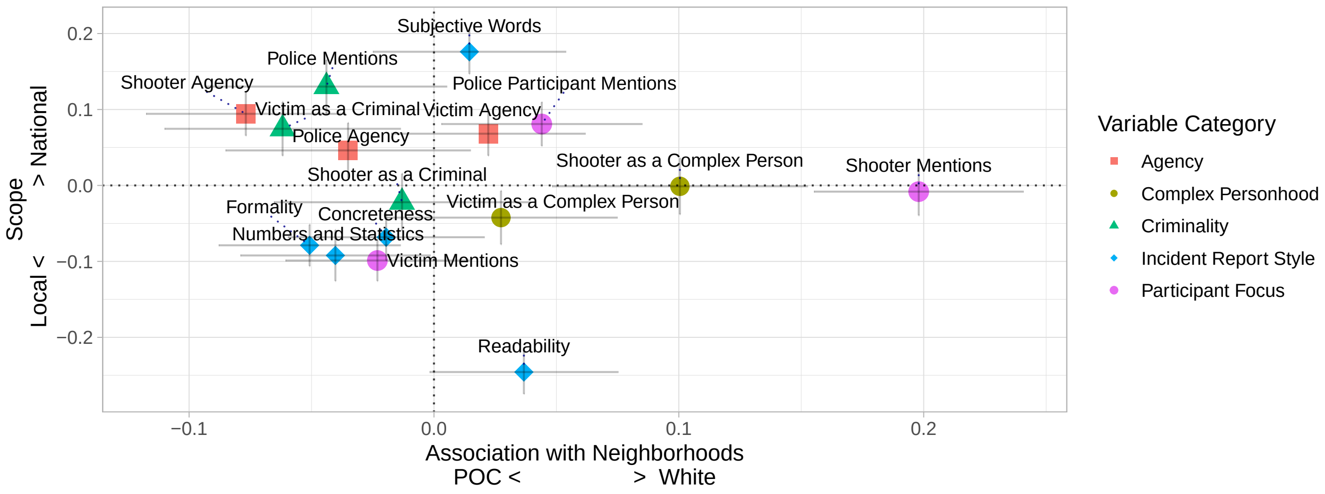

Fig. S2. Disproportionate occurrence of framing and linguistic features by neighborhood racial composition and news source scope. Points are regression coefficients of each z-scored framing feature predicting race binary and source scope including controls. Error bars represent 95% confidence intervals.

Table S5. Average accuracy of predictions of race based on articles using different LLMs. The dataset uses 8043 pairs of articles from each group (Majority white and Majority POC), where each pair is matched on incident features. Accuracy is the average accuracy using five-fold cross-validation.

Model	Epochs	Max tokens	Average Accuracy
Unigram/Bigram SVC		None	0.7119
Deberta-base	3	500	0.7307
ModernBERT-base	5	1000	0.7408
ModernBERT-base	3	1000	0.7487
ModernBERT-large	3	1000	0.7586

Table S6. Regression predicting four coverage features based on incident features and a neighborhood racial composition.

	Dependent variable:			
	OLS		*Logistic*	
	Article Count	Article Length	National Coverage	2+ Articles
White Neighborhood	−0.200	−7.797	−0.046	0.026
Named Participants	0.030	85.343***	0.092***	0.170***
Mass Shooting	1.073	86.921***	0.134***	0.105***
Hate Crime	10.802***	256.648***	0.408***	0.364***
Child Involved	1.289*	57.594***	0.119***	0.161***
School Related	6.603***	152.069***	0.173**	0.276***
Gang Related	−0.359	69.752***	0.016	0.092***
Drug Involved	−0.611	−18.647	0.001	−0.035
Officer Involved	4.601***	197.697***	0.230***	0.330***
Suicide	0.571	21.716	0.100***	0.025
Domestic Violence	−0.832*	−20.055	−0.013	0.011
Home Invasion	−0.498	−10.578	−0.006	0.047
Accident	0.838	−19.584	0.032	0.025
Assault Weapon	5.264***	39.538	0.069*	0.071*
No Traditional Victims	−1.867**	−52.041***	−0.122***	−0.160***
Injury Only	−1.227***	−31.092***	−0.055***	−0.093***
4+ Deaths	12.918***	107.010**	0.141**	0.186***
White * Named Participants	0.597	40.518*	0.058*	0.045
White * Mass Shooting	2.672**	35.220	0.123***	0.109**
White * Hate Crime	3.568	67.985	−0.122	−0.057
White * Child	−1.409	−59.164*	0.033	−0.080*
White * School	7.890***	118.799*	0.026	−0.040
White * Gang	−0.733	−37.852	−0.047	−0.074
White * Drug	0.008	73.656**	−0.063	−0.008
White * Officer	−2.784***	−62.305***	−0.038	−0.102***
White * Suicide	−1.624*	20.620	−0.066*	−0.021
White * Domestic Violence	−0.169	0.776	0.004	0.008
White * Home Invasion	0.437	−20.023	0.0001	−0.035
White * Accident	−0.961	−9.729	−0.073*	−0.043
White * Assault Weapon	2.108	21.600	0.068	0.013
White * No Traditional Victims	0.159	−46.162*	−0.024	−0.078*
White * Injury Only	0.341	−1.780	−0.005	−0.005
White * 4+ Deaths	0.912	18.294	0.008	0.014
Constant	1.734***	318.708***	0.118***	0.081***
Observations	15,249	15,249	15,249	15,249
R^2	0.075	0.088		
Adjusted R^2	0.073	0.086		
Log Likelihood			−7,989.498	−8,651.409
Akaike Inf. Crit.			16,047.000	17,370.820
Residual Std. Error (df = 15215)	10.804	288.192		
F Statistic (df = 33; 15215)	37.395***	44.632***		
Note:			*p<0.05; **p<0.01; ***p<0.001	

Table S7. Results from regression models predicting incident coverage based on the demographic features of the neighborhood where the incident occurred.

	Dependent variable:	
	OLS	*Logistic*
	Article Count	Any Articles
Adults who graduated high school	−0.002	0.001*
Families below poverty line	0.035**	0.001*
Unemployment	0.010	−0.003**
Female Heads of Household	−0.005	0.0002
Population under age 18	−0.022	−0.002***
Individuals on Public assistance	−0.004	0.002*
Income	1.563***	0.084***
Population Density	−0.062	−0.005*
Constant	−13.703***	−0.626***
Observations	15,249	15,249
R^2	0.002	
Adjusted R^2	0.002	
Log Likelihood		−9,500.292
Akaike Inf. Crit.		19,018.580
Residual Std. Error	11.211 (df = 15240)	
F Statistic	4.700*** (df = 8; 15240)	
Note:	*p<0.05; **p<0.01; ***p<0.001	

Table S8. Regression predicting two coverage features based on incidents features and a larger set of neighborhood features.

		Dependent variable:	
		OLS Article Count	*Logistic* 2+ Articles
Neighborhood Features	White Neighborhood	−0.587	−0.001
	Adults who graduated high school	−1.597*	−0.019
	Families below poverty line	−0.166	0.010
	Unemployment	0.542	−0.031
	Female Heads of Household	−0.955	−0.034
	Population under age 18	2.011	0.005
	Individuals on Public assistance	0.156	−0.081**
	Income	0.330	−0.009
	Population Density	0.966	0.027
Incident Features	Named Participants	0.070	0.177***
	Mass Shooting	1.069	0.100***
	Hate Crime	10.938***	0.379***
	Child Involved	1.381**	0.171***
	School Related	6.438***	0.264***
	Gang Related	−0.308	0.099***
	Drug Involved	−0.560	−0.026
	Officer Involved	4.607***	0.333***
	Suicide	0.460	0.021
	Domestic Violence	−0.845*	0.013
	Home Invasion	−0.559	0.051
	Accident	0.803	0.020
	Assault Weapon	5.272***	0.074*
	No Traditional Victims	−1.918**	−0.162***
	Injury Only	−1.214***	−0.091***
	4+ Deaths	12.883***	0.187***
Interactions	White * Named Participants	0.003	0.0005
	White * Mass Shooting	0.031**	0.001*
	White * Hate Crime	0.007	−0.002
	White * Child	0.008	0.002***
	White * School	−0.014	−0.002***
	White * Gang	−0.011	0.002
	White * Drug	1.542***	0.090***
	White * Officer	−0.016	0.004
	White * Suicide	0.689	0.057*
	White * Domestic Violence	2.655**	0.103**
	White * Home Invasion	3.580	−0.062
	White * Accident	−1.479	−0.087*
	White * Assault Weapon	8.189***	−0.021
	White * No Traditional Victims	−0.991	−0.098
	White * Injury Only	0.081	−0.003
	White * 4+ Deaths	−2.743***	−0.098***
	Constant	−15.345***	−0.974***
	Observations	15,249	15,249
	R^2	0.077	
	Adjusted R^2	0.074	
	Log Likelihood		−8,579.846
	Akaike Inf. Crit.		17,243.690
	Residual Std. Error	10.796 (df = 15207)	
	F Statistic	30.916*** (df = 41; 15207)	
	Note:		*p<0.05; **p<0.01; ***p<0.001

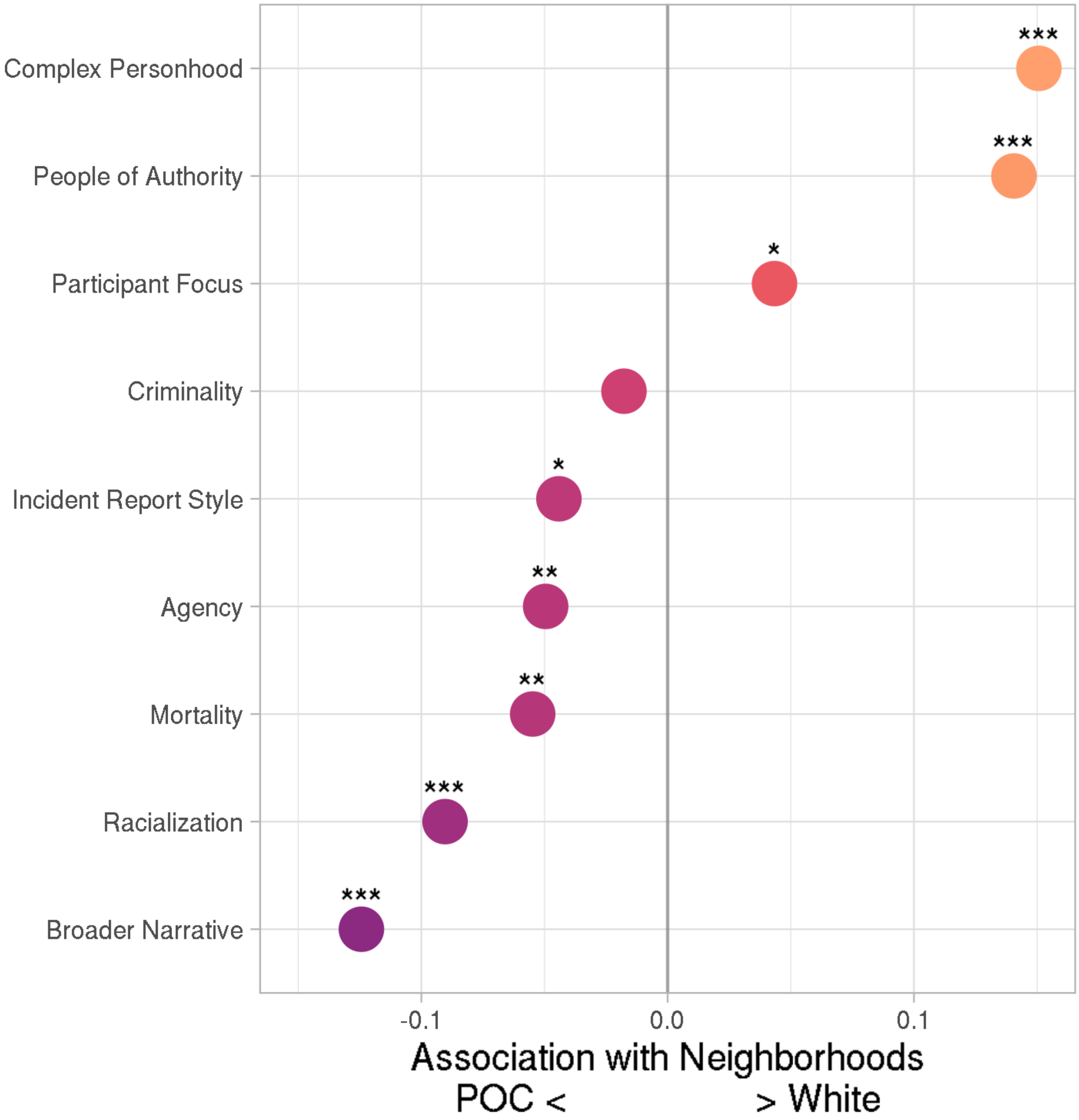

Fig. S3. Disproportionate occurrence of grouped content and framing features by neighborhood racial composition. Points are regression coefficients the grouped features predicting the racial composition group including controls. * is p<0.05.

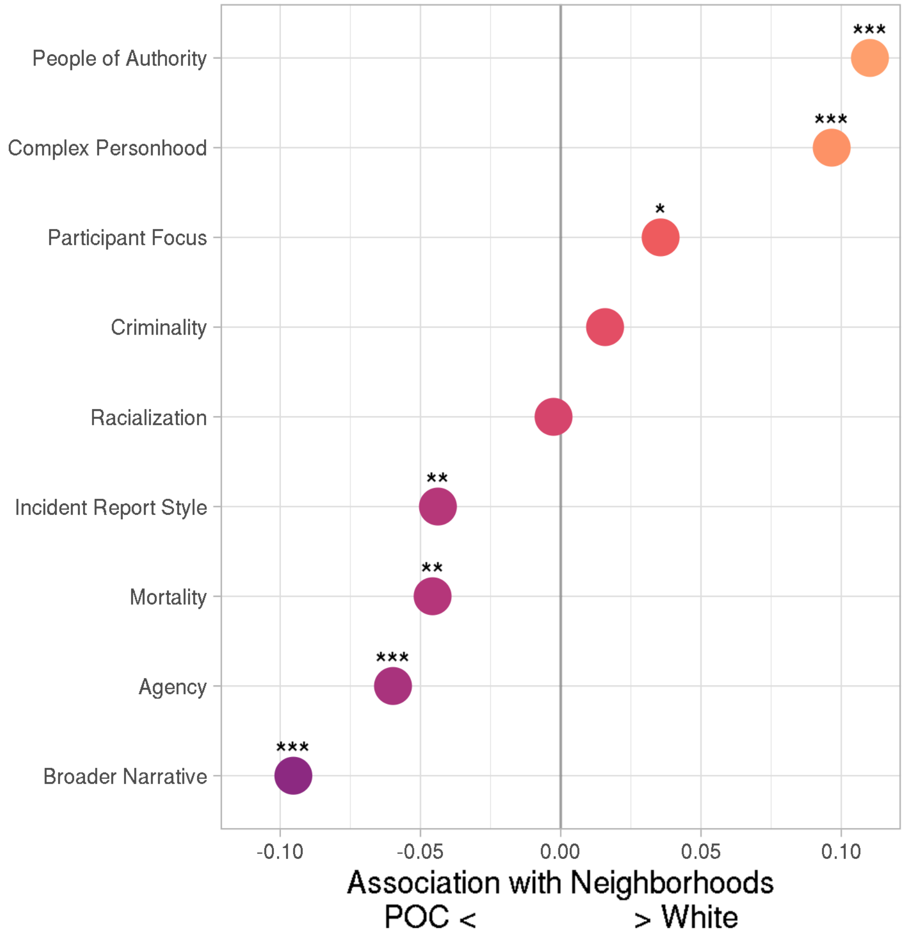

Fig. S4. Grouped ontological categories using a simple majority for the race variable, instead of the 60% supermajority of non-Hispanic white residents used in the main paper

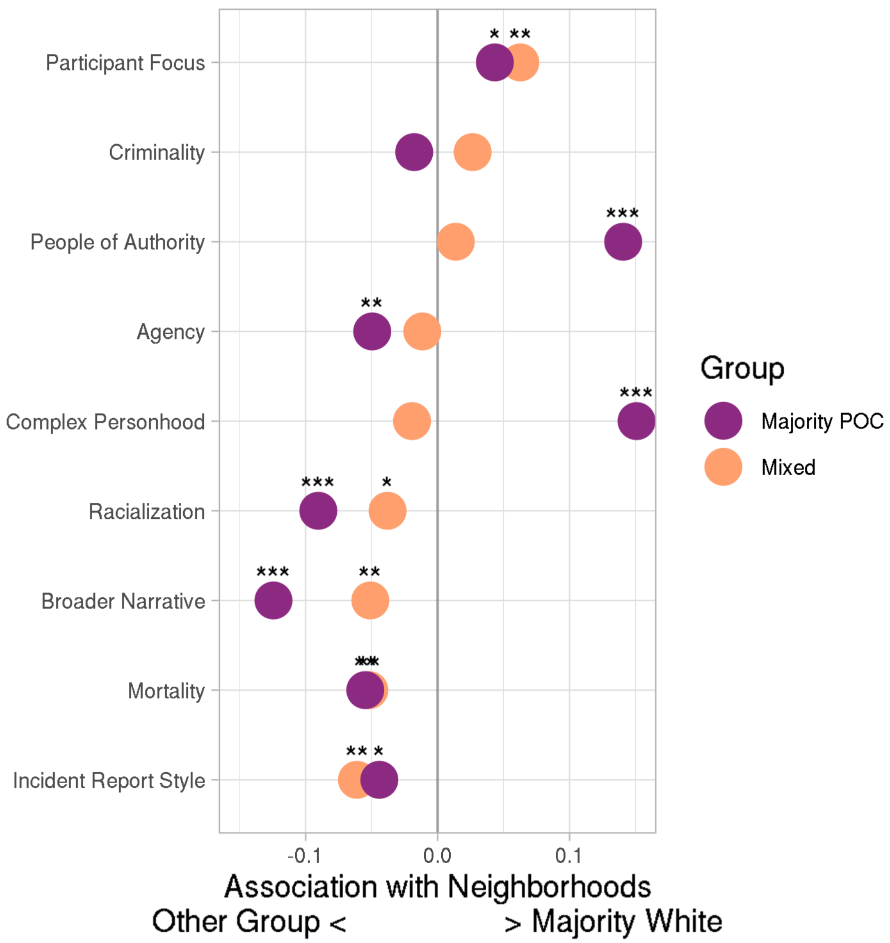

Fig. S5. Grouped ontological categories comparing Mixed neighborhoods (40-60% white) and Majority POC neighborhoods to Majority white neighborhoods

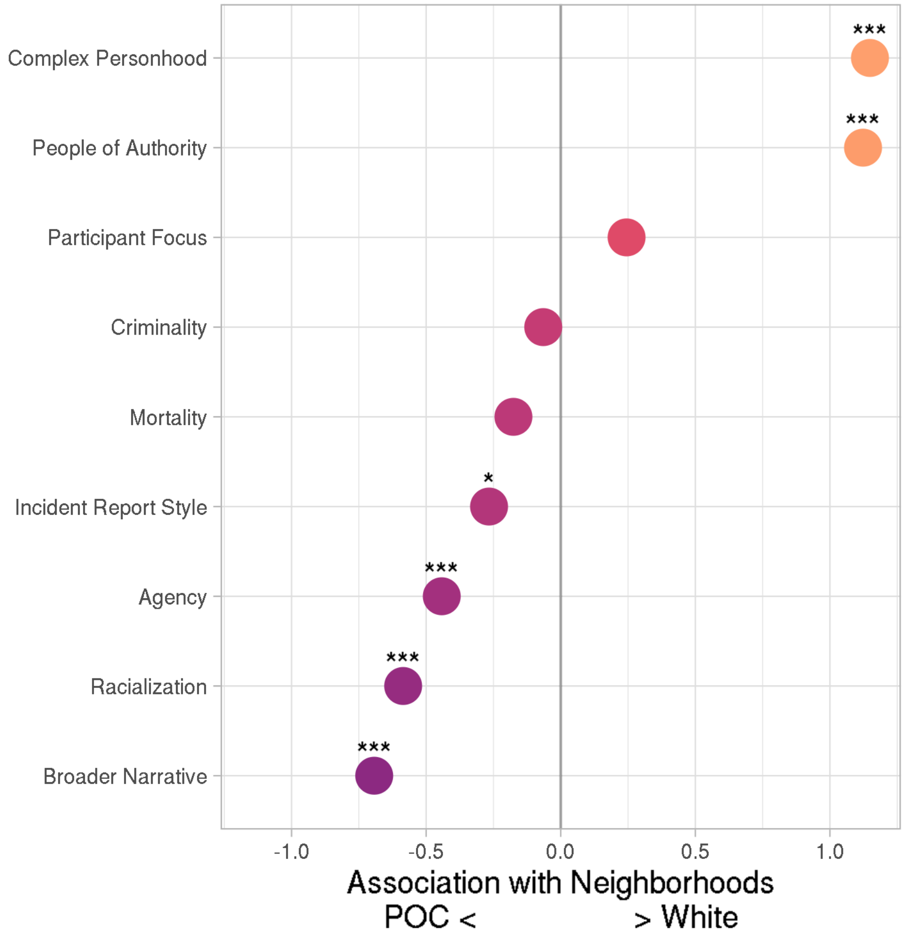

Fig. S6. Grouped ontological categories showing results of a linear regression predicting neighborhood racial composition as a continuous variable (% white).

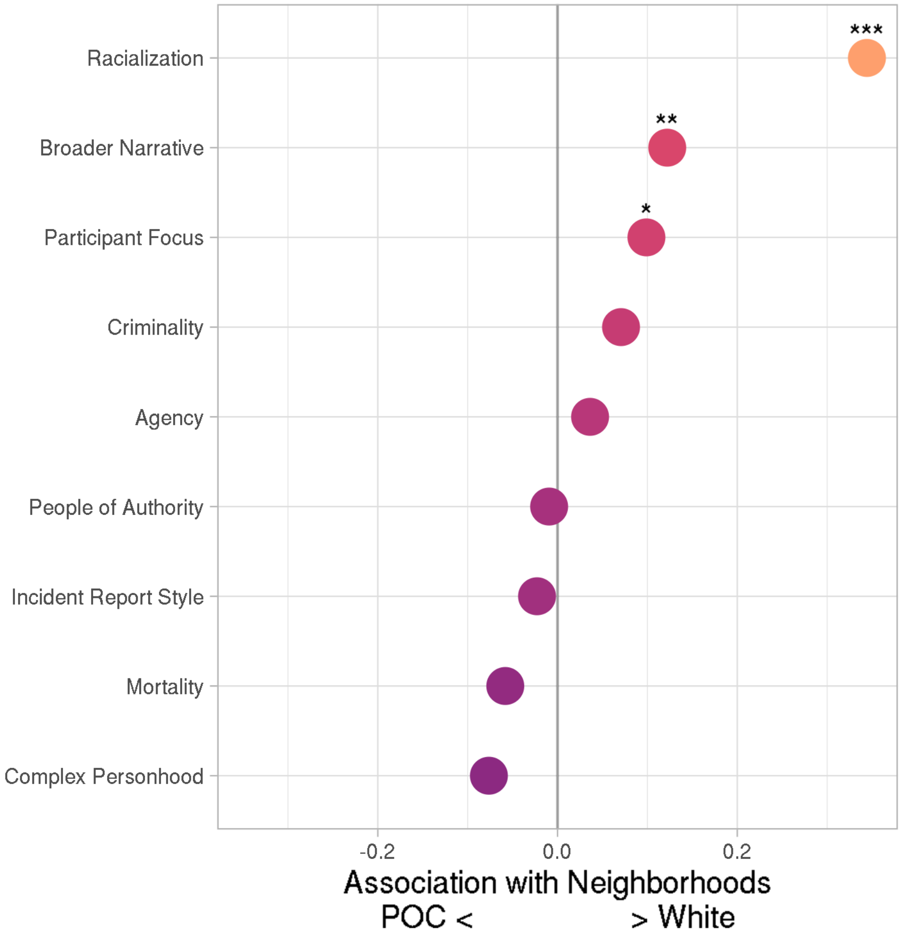

Fig. S7. Grouped ontological categories using whether the article mentioned only non-white racial descriptors, or described anyone as 'white'.

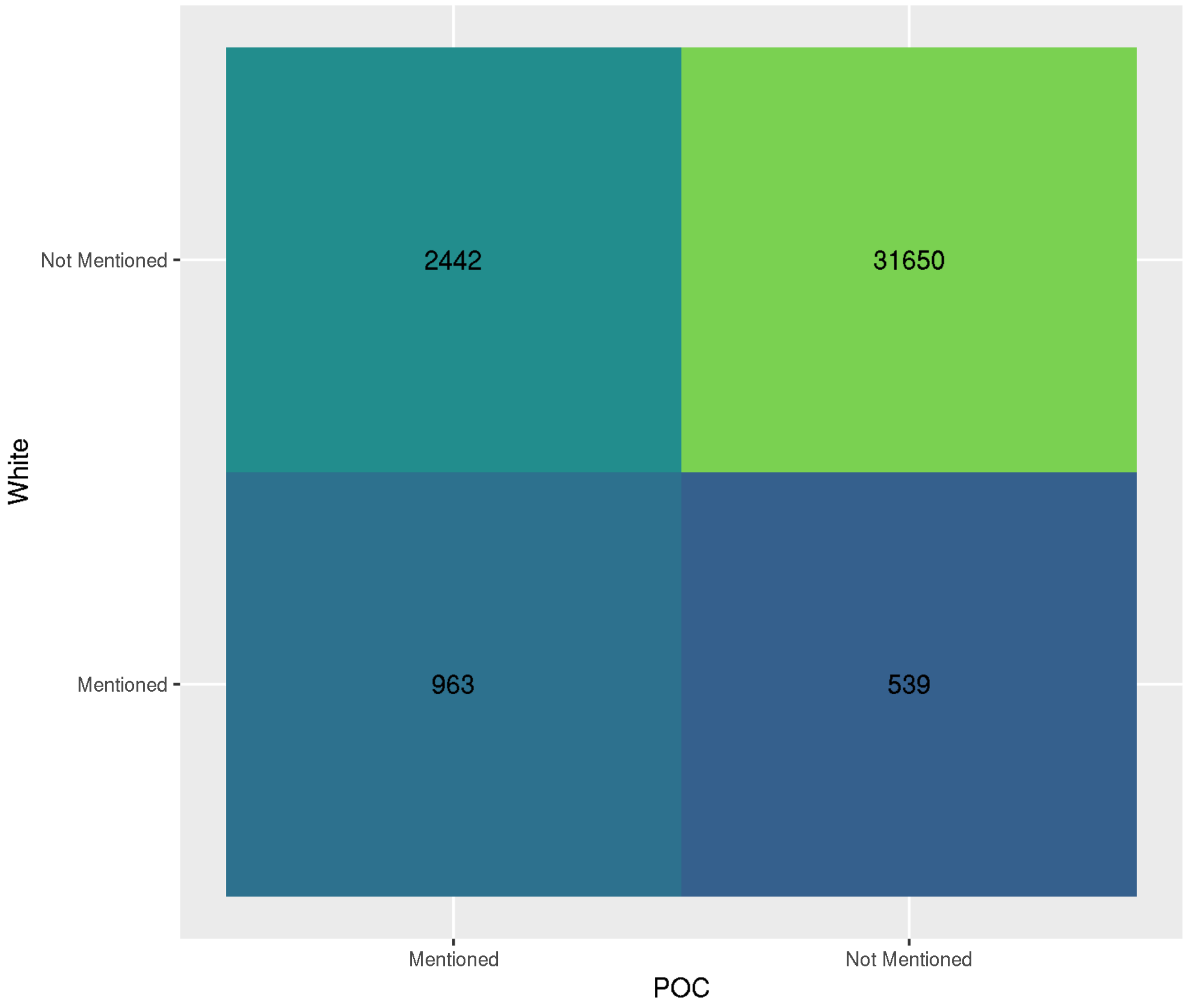

Fig. S8. Frequency table of the number of articles that explicitly mention the race of individuals (white and POC) in our dataset

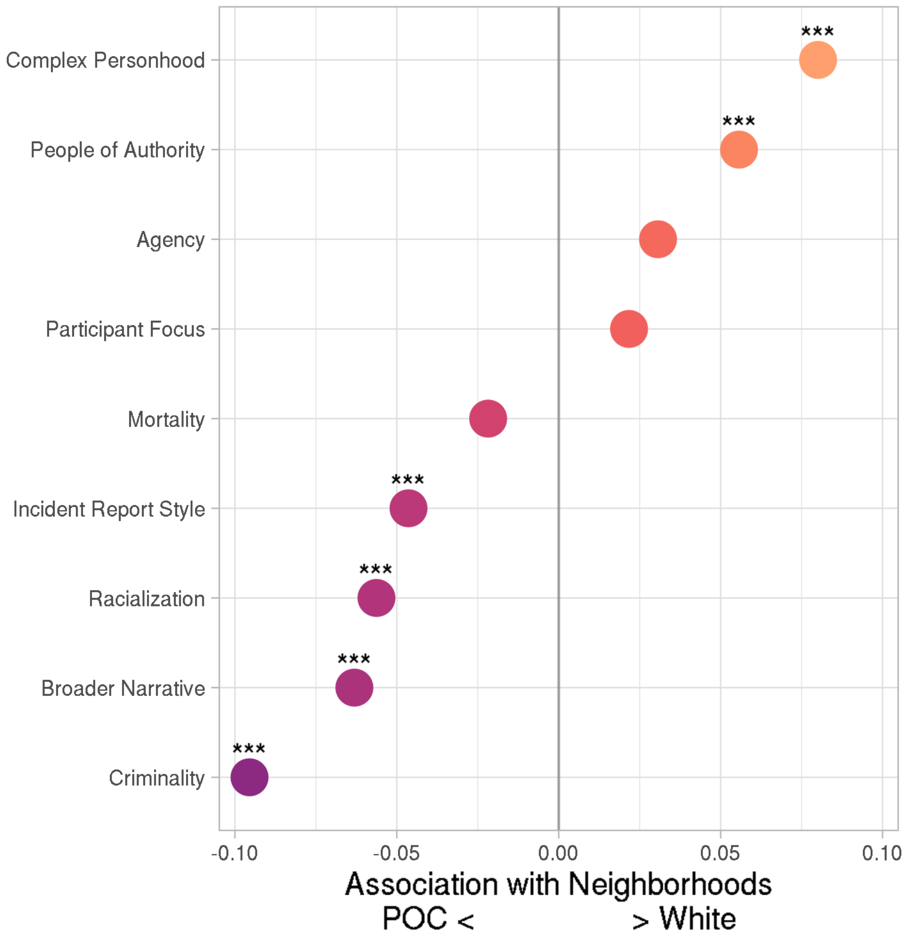

Fig. S9. Grouped ontological categories using coarsened exact matching to predict each ontology feature based on incident characteristics and matched on neighborhood characteristics

Table S9

	Dependent variable:
	Majority White
Incident Report Style	0.154*
Participant Focus	0.030
Agency	−0.205**
People of Authority	0.491***
Complex Personhood	0.155*
Criminality	0.332***
Mortality	−0.117*
Racialization	0.021
Broader Narrative	−0.386***
Medium Density	−2.022***
High Density	−3.141***
Hate Crime	0.657***
Child Involved	−0.386***
School Related	0.772***
Gang Related	−0.497***
Drug Involved	0.543***
Officer Involved	−0.324***
Suicide	−0.166**
Domestic Violence	0.405***
Home Invasion	0.257*
Accident	−0.581***
Assault Weapon	0.198**
Named Victims	0.208**
Named Shooters	0.678***
Mass Shooting	0.371***
4+ Deaths	0.321***
Injury Only	0.483***
Median Income	0.359***
Length	0.00001
Incident Report Style * Medium Density	−0.160*
Incident Report Style * High Density	−0.258***
Participant Focus * Medium Density	−0.029
Participant Focus * High Density	0.157*
Agency * Medium Density	0.117
Agency * High Density	0.233***
People of Authority * Medium Density	−0.304***
People of Authority * High Density	−0.461***
Complex Personhood * Medium Density	0.069
Complex Personhood * High Density	−0.162*
Criminality * Medium Density	−0.376***
Criminality * High Density	−0.430***
Mortality * Medium Density	0.095
Mortality * High Density	0.050
Racialization * Medium Density	−0.244*
Racialization * High Density	0.059
Broader Narrative * Medium Density	0.283***
Broader Narrative * High Density	0.238**
Constant	0.606***
Observations	31,365
Log Likelihood	−11,210.730
Akaike Inf. Crit.	22,519.470
Note:	*p<0.05; **p<0.01; ***p<0.001

References

1. AN Gobaud, et al., Assessing the Gun Violence Archive as an Epidemiologic Data Source for Community Firearm Violence in 4 US Cities. *JAMA Netw. Open* **6**, e2316545 (2023).
2. M Sap, MC Prasettio, A Holtzman, H Rashkin, Y Choi, Connotation Frames of Power and Agency in Modern Films in *Proceedings of the 2017 Conference on Empirical Methods in Natural Language Processing.* (Association for Computational Linguistics, Copenhagen, Denmark), pp. 2329–2334 (2017).
3. D Card, et al., Computational analysis of 140 years of US political speeches reveals more positive but increasingly polarized framing of immigration. *Proc. Natl. Acad. Sci.* **119**, e2120510119 (2022) Publisher: Proceedings of the National Academy of Sciences.
4. T Wilson, J Wiebe, P Hoffmann, Recognizing Contextual Polarity in Phrase-Level Sentiment Analysis in *Proceedings of Human Language Technology Conference and Conference on Empirical Methods in Natural Language Processing*, eds. R Mooney, C Brew, LF Chien, K Kirchhoff. (Association for Computational Linguistics, Vancouver, British Columbia, Canada), pp. 347–354 (2005).
5. JP Kincaid, RP Fishburne Jr, RL Rogers, BS Chissom, Derivation of new readability formulas (automated readability index, fog count and flesch reading ease formula) for navy enlisted personnel, Technical report (1975).
6. M Brysbaert, AB Warriner, V Kuperman, Concreteness ratings for 40 thousand generally known English word lemmas. *Behav. Res. Methods* **46**, 904–911 (2014).
7. RD Peterson, LJ Krivo, Racial Segregation, the Concentration of Disadvantage, and Black and White Homicide Victimization. *Sociol. Forum* **14**, 465–493 (1999) Company: Springer Distributor: Springer Institution: Springer Label: Springer Number: 3 Publisher: Kluwer Academic Publishers-Plenum Publishers.
8. JH Beard, et al., Systematic disparities in reporting on community firearm violence on local television news in Philadelphia, PA, USA. *Prev. Medicine Reports* **42**, 102739 (2024).
9. K White, F Stuart, SL Morrissey, Whose Lives Matter? Race, Space, and the Devaluation of Homicide Victims in Minority Communities. *Sociol. Race Ethn.* **7**, 333–349 (2021) Publisher: SAGE Publications Inc.
10. M Sen, O Wasow, Race as a Bundle of Sticks: Designs that Estimate Effects of Seemingly Immutable Characteristics. *Annu. Rev. Polit. Sci.* **19**, 499–522 (2016).
